# Machine Learning-Driven Consensus Modeling for Activity Ranking and Chemical Landscape Analysis of HIV-1 Inhibitors

**DOI:** 10.3390/ph18050714

**Published:** 2025-05-13

**Authors:** Danishuddin, Md Azizul Haque, Geet Madhukar, Qazi Mohammad Sajid Jamal, Jong-Joo Kim, Khurshid Ahmad

**Affiliations:** 1 Department of Biotechnology, Yeungnam University, Gyeongsan 38541, Republic of Korea; danish23@yu.ac.kr (D.); azizul@ynu.ac.kr (M.A.H.); 2 Department of Molecular, Cellular and Biomedical Sciences, University of New Hampshire, Durham, NH 03824, USA; geet.madhukar@unh.edu; 3 Department of Health Informatics, College of Applied Medical Sciences, Qassim University, Buraydah 51452, Saudi Arabia; m.quazi@qu.edu.sa

**Keywords:** machine learning, consensus modeling, HIV integrase, scaffolds, activity landscape

## Abstract

**Background/Objective:** This study aimed to develop a predictive model to classify and rank highly active compounds that inhibit HIV-1 integrase (IN). **Methods**: A total of 2271 potential HIV-1 inhibitors were selected from the ChEMBL database. The most relevant molecular descriptors were identified using a hybrid GA-SVM-RFE approach. Predictive models were built using Random Forest (RF), eXtreme Gradient Boosting (XGBoost), Support Vector Machines (SVM), and Multi-Layer Perceptron (MLP). The models underwent a comprehensive evaluation employing calibration, Y-randomization, and Net Gain methodologies. **Results**: The four models demonstrated intense calibration, achieving an accuracy greater than 0.88 and an area under the curve (AUC) exceeding 0.90. Net Gain at a high probability threshold indicates that the models are both effective and highly selective, ensuring more reliable predictions with greater confidence. Additionally, we combine the predictions of multiple individual models by using majority voting to determine the final prediction for each compound. The Rank Score (weighted sum) serves as a confidence indicator for the consensus prediction, with the majority of highly active compounds identified through high scores in both the 2D descriptors and ECFP4-based models, highlighting the models’ effectiveness in predicting potent inhibitors. Furthermore, cluster analysis identified significant classes associated with vigorous biological activity. **Conclusions:** Some clusters were found to be enriched in highly potent compounds while maintaining moderate scaffold diversity, making them promising candidates for exploring unique chemical spaces and identifying novel lead compounds. Overall, this study provides valuable insights into predicting integrase binders, thereby enhancing the accuracy of predictive models.

## 1. Introduction

Human immunodeficiency virus (HIV), the causative agent of AIDS, now infects millions of people worldwide [1,2]. Despite significant advances in medical research, there are still no vaccines or curative treatments for HIV-1. As a result, antiretroviral therapy (ART) remains the cornerstone of HIV-1 management, focusing on suppressing viral replication, alleviating symptoms, and delaying progression to Acquired Immunodeficiency Syndrome (AIDS) [3]. Effective clinical regimens often employ combinations of antiretroviral (ARV) drugs to enhance antiviral efficacy and minimize the emergence of drug-resistant strains [4,5].

The integrase (IN) enzyme plays a pivotal role in the HIV-1 replication cycle, facilitating the integration of viral DNA into the host genome, which is critical to establishing infection [6,7]. This unique function makes the integrase enzyme an attractive target for therapeutic intervention. Integrase inhibitors block this integration process, halting viral replication and limiting disease progression [8,9]. Over the years, multiple classes of ARV drugs have been developed, targeting various stages of the HIV-1 life cycle [10]. These include reverse transcriptase, protease fusion, and integrase inhibitors [11].

The development of integrase strand transfer inhibitors (INSTIs) represents a significant advancement in HIV-1 treatment [12]. These inhibitors, including FDA-approved drugs such as raltegravir, elvitegravir, and dolutegravir, have demonstrated high potency, favorable safety profiles, and a reduced risk of cross-resistance with other ARV drug classes [13]. As a result, INSTIs have become an integral part of first-line HIV-1 therapy, offering a promising approach to long-term viral suppression and improved patient outcomes [14]. Research into integrase inhibitors continues to evolve, with efforts focusing on enhancing drug potency, overcoming resistance mutations, and improving pharmacokinetics to simplify treatment regimens [15]. By targeting the integrase enzyme, medicinal chemistry researchers aim to develop more effective therapies that address the ongoing challenges posed by HIV-1 [16].

In recent years, computational techniques, particularly machine learning (ML), have gained prominence in drug discovery and development [17,18]. ML models may assess chemical structures, predict biological activities, and provide insights into structure–activity relationships (SAR). By leveraging extensive datasets and advanced algorithms, ML models can analyze chemical structures, predict biological activities, and provide insights into structure–activity relationships (SAR) [19]. These attributes are crucial for HIV-1 integrase inhibitors, necessitating precision, efficiency, and innovation. In contrast to traditional QSAR models that employ linear equations to associate molecular descriptors with biological activities, machine learning facilitates the development of non-linear QSAR models, enhancing predictive performance [20].

While numerous studies have explored IN-ligand recognition, inhibition mechanisms, and molecular modifications, two critical scientific challenges remain unresolved: (1) Is there an effective classification method to evaluate the activity of the reported IN inhibitors? (2) How can inhibitors be effectively modified by identifying the key structural features influencing molecular activity? We developed consensus predictive models for identifying promising IN-potent inhibitors to address these challenges. These models can accelerate the discovery of potent compounds, guide the rational design of next-generation drugs, and support personalized treatment strategies. We also analyzed the activity landscape of the inhibitors and performed a chemoinformatics-based characterization of the SAR of a dataset. This analysis focused on three specific objectives: (a) characterizing the structural diversity and distribution of the dataset within the chemical space; (b) conducting a descriptive SAR analysis; (c) activity divergence and scaffold analyses. The structure-based interpretation of the SAR provided key insights into the molecular mechanisms underlying the inhibition of active molecules. This analysis also suggested structural modifications to lead compounds, supporting the continued development of IN as a potential inhibitor.

## 2. Results

### 2.1. Selection of Molecular Descriptors and Chemical Space Analysis

A total of 1652 molecular descriptors were calculated using the RDKit and PaDEL frameworks. These descriptors were then refined to remove constant or zero values. Subsequently, the descriptors were processed using a hybrid feature selection approach that combined GA, SVM, and RFE. After a comprehensive analysis, 44 of the most significant descriptors were identified and selected (Supplementary Table S1). These descriptors include Autocorrelation, Barysz Matrix, ALogP2, Carbon Types, Chi Chain, Constitutional, Atom-Type Electrotopological State, Extended Topochemical Atom, Weighted Path, and others. These descriptors were crucial in designing enzyme inhibitors by capturing key molecular properties essential for binding and activity modulation. For instance, ALogP2 aids in optimizing lipophilicity and enhancing cell permeability, while electrotopological state descriptors help to identify functional groups that strengthen binding. They are also helpful in recognizing functional groups that improve chelation with Mg^2^⁺ ions, a critical mechanism for integrase inhibition. Additionally, weighted path and autocorrelation descriptors provide insights into molecular conformation and electronic distribution, facilitating π–π stacking and hydrogen bonding interactions with active site residues.

This study characterized the chemical space using the selected descriptors and fingerprints employed for model development. To compare the property distribution between the training and test sets, principal component analysis (PCA) was conducted. The molecules were projected into a 2D space, demonstrating a similar distribution of chemical space across the training and test sets (Supplementary Figure S1a,b). Compounds within the AD are expected to have reliable predictions, whereas those outside may exhibit lower prediction confidence. We analyzed the test set compounds to evaluate the AD of our predictive model. The test set of compounds was assessed based on their distances. The distribution of compounds in the test set is primarily confined within the chemical space of the training set, indicating that the evaluation of the developed model using these test compounds is viable.

### 2.2. Model Development and Evaluation

**Figure 1:**
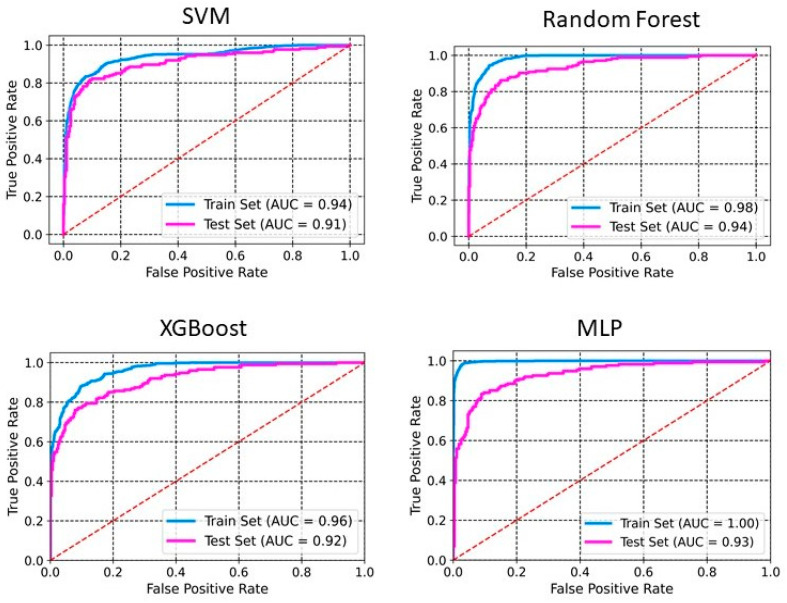
ROC curves for PaDEL+RDKit 2D descriptors. The ROC curve illustrates the model’s ability to discriminate between classes by plotting the true positive rate against the false positive rate.

**Figure 2:**
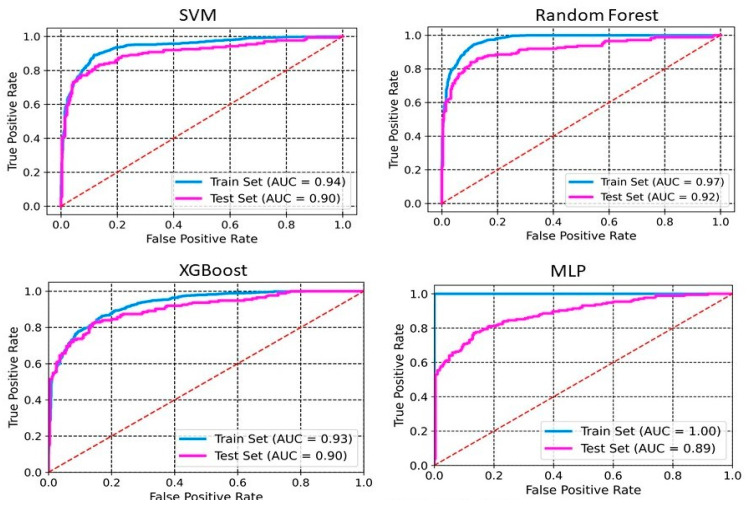
ROC curves for ECFP4 fingerprints. The ROC curve illustrates the model’s ability to discriminate between classes by plotting the true positive rate against the false positive rate.

The models were built using four different methods: SVM, RF, XGBoost, and MLP. Table 1 and Table 2 summarize the performance of the developed classification models based on selected 2D descriptors and ECFP4 fingerprints, respectively. XGBoost achieves an accuracy of 0.98 and 0.88 on the training set and the test set, respectively (Table 1). The sensitivity, specificity, precision, and F1 scores for this model on the test set are 0.81, 0.93, 0.87, and 0.84, respectively. Whereas SVM and MLP both achieve an accuracy of 0.87 on the test set. RF performs with an accuracy of 0.91 and 0.86 on training and test sets, respectively. The sensitivity, specificity, precision, and F1 scores for this model on the test set are 0.72, 0.95, 0.90, and 0.80, respectively. The ROC-AUC curve of these models demonstrates a robust model with a good balance between detecting positive and negative cases (Figure 1 and Figure 2). The AUCs of SVM, RF, XGBoost, and MLP are 0.91, 0.94, 0.92, and 0.93, respectively (Figure 1). The RF model, utilizing ECFP4 fingerprints with 1024 bits, achieved an accuracy of 0.85 on the test set. It demonstrated a sensitivity of 0.78, a specificity of 0.92, a precision of 0.86, an F1 score of 0.82, and an AUC of 0.92. The performance of the XGBoost model based on the ECFP4 fingerprint obtained an accuracy of 0.90 and 0.85 on the training and test sets, respectively. The sensitivity, specificity, precision, and F1 scores for this model on the test set are 0.77, 0.91, 0.85, and 0.80, respectively. Meanwhile, SVM and MLP perform with accuracies of 0.86 and 0.84, respectively. Among all four models, MLP exhibited the lowest sensitivity at 0.66 and F1 score at 0.74 (Table 2). The AUC values for SVM, RF, XGBoost, and MLP using ECFP4 fingerprints are 0.90, 0.97, 0.93, and 0.89, respectively (Figure 2).

The Net Gain was analyzed across different probability thresholds to evaluate model performance. Based on this analysis, at a 0.90 probability threshold, the Net Gain was observed as 0.67 for SVM, whereas it was 0.98 and 0.86 for RF and XGBoost, respectively. At 0.85, the Net Gain values were 0.70, 0.88, and 0.79 for SVM, RF, and XGBoost, respectively. RF consistently achieved the highest Net Gain across different probability thresholds, comparing all four models, indicating its superior predictive performance. The Net Gain for RF was observed to be 0.80, 0.92, 0.95, 0.92, 0.88, and 0.98 at probability thresholds of 0.50, 0.70, 0.75, 0.80, 0.85, and 0.90, respectively. The increasing Net Gain as the probability threshold rises suggests that this model makes more beneficial and selective predictions at higher confidence levels. It demonstrates the model’s ability to maximize true positives while minimizing false positives. It reflects the model’s ability to maximize true positives while reducing false positives. For MLP, the Net Gain was found to be low compared to other models. For the ECFP4-based RF model, the Net Gain was found to be 0.91 at a probability of 0.75 and 0.85, indicating that it is most effective at these thresholds in balancing true and false positives. However, at a higher probability of 0.90, its Net Gain (0.85) decreases, and its predictive advantage diminishes as stricter confidence levels are applied. In contrast, the SVM and XGBoost models based on ECFP4 achieved a higher Net Gain of 0.83 at a probability of 0.90 and 0.94 at a probability of 0.80, respectively. XGBoost outperforms RF and SVM in terms of Net Gain, suggesting that it may be the most reliable model for ECFP4-based predictions, particularly when aiming for a strong predictive benefit with a relatively high probability threshold. Notably, most predicted true positive compounds at a probability threshold > 0.90 exhibited high potency. However, some false positive predictions also had IC_50_ values below 10μM, which is generally considered an acceptable threshold in drug discovery. This highlights the importance of incorporating Net Gain analysis to assess the predictive reliability of machine learning models, particularly in identifying potentially active compounds. These findings support the robustness and true predictive power of the developed models for HIV-1.

$$NetGain=\frac{TP}{N}-\left(\frac{FP}{N}\times w\right)$$

where $w=\frac{Pr}{1-Pr}$; Pr = Probability cutoff; N = number of compounds.

### 2.3. Calibration Plot

**Figure 3:**
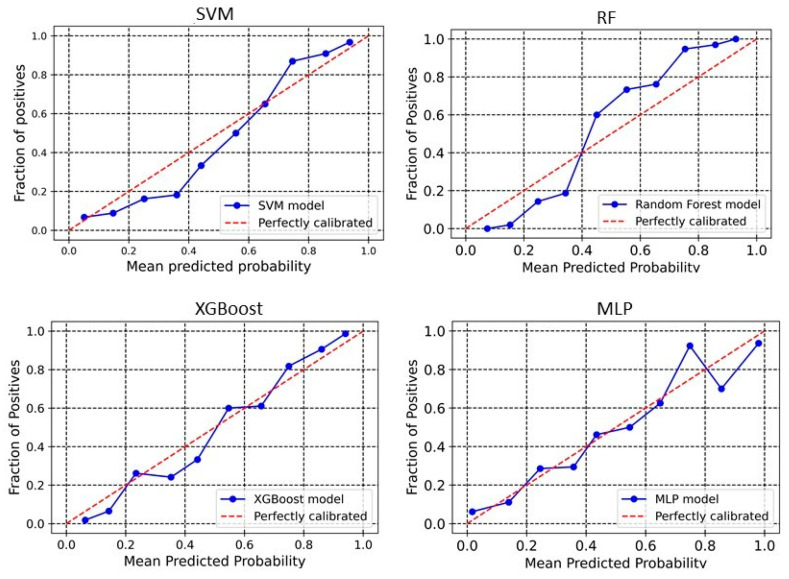
Calibration curve for PaDEL+RDKit descriptors-based model. The calibration curve evaluates the agreement between predicted probabilities and observed outcomes by plotting the mean predicted probabilities at different quantiles of the true probabilities, with the diagonal line indicating perfect calibration.

**Figure 4:**
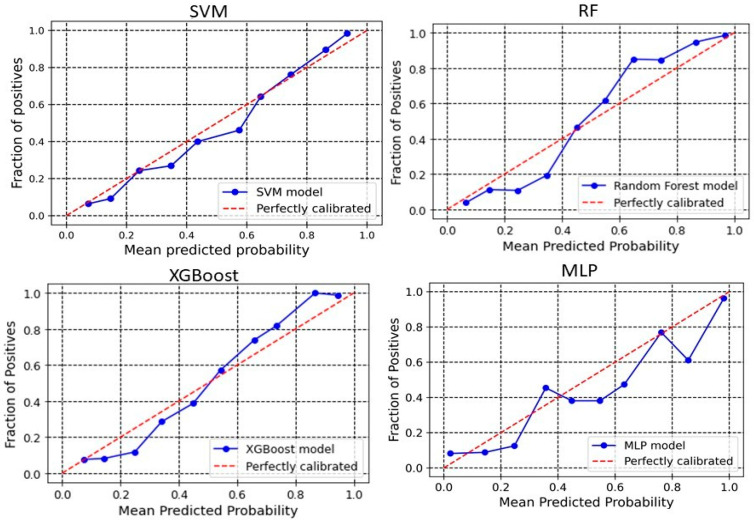
Calibration curve for ECFPs model. The calibration curve evaluates the agreement between predicted probabilities and observed outcomes by plotting the mean predicted probabilities at different quantiles of the true probabilities, with the diagonal line indicating perfect calibration.

The calibration plot reveals the relationship between the predicted probabilities of the developed models and the actual observed outcomes. Analyzing calibration across more bins provides deeper insights into each model’s performance. The significance of calibration analysis lies in its ability to provide more than accuracy metrics. It evaluates how well the predicted probabilities correspond to actual outcomes, which is crucial for models used in high-stakes decisions. Calibration analysis ensures accurate classifications and meaningful probabilities, enhancing decision-making processes. Here, we perform the calibration analyses of the models. The outcomes of these analyses have been shown in Figure 3 and Figure 4 for 2D descriptors and ECFP4 fingerprints models, respectively. The developed models demonstrate good calibration, particularly at higher probability bins (Except MLP), as the points align closely with the diagonal “perfectly calibrated” line in these regions. For SVM, the deviation is seen in the lower and middle bins. At 0.376, the observed fraction is only 0.186, showing under-calibration, and at 0.431, the observed fraction improves to 0.373. However, higher bins like 0.857 and 0.937 demonstrate better alignment with an observed fraction of 0.9091 and 0.954, respectively, indicating that SVM performs better at high probabilities (Figure 3). For XGBoost, the calibration curve is consistently close to the ideal diagonal line across higher bins (Figure 3). For example, at a predicted probability of 0.860, the observed fraction is 0.892, and at 0.940, the observed fraction is 0.96. In the RF model, poor calibration is evident in the lower bins. For instance, at a predicted probability of 0.152, the observed fraction is only 0.019, highlighting underestimation. However, higher bins like 0.754 (observed fraction 0.947) and 0.928 (observed fraction 1.0) show improved alignment, suggesting that RF performs better for higher confidence predictions (Figure 3). The MLP model demonstrates better calibration in lower and mid bins. For instance, at 0.546, the observed fraction aligns at 0.5. However, at the upper bins, the observed fraction shows a sharp deviation, suggesting over-calibration in the higher bins. In the case of the ECFP4-based SVM model, the predicted probabilities around 0.24–0.64 are generally well-aligned with observed values, meaning the model’s probability estimates are reliable in this range. For probabilities 0.34–0.57, the predicted probability is higher than the observed fraction of positives (Figure 4). This suggests that in this range, the model is slightly overconfident, predicting a higher likelihood of positives than observed. In the higher probability bins (0.74–0.93), the model slightly underestimates the fraction of positives. XGBoost model overestimates positive outcomes at low probabilities (0.14 and 0.24 probability bins), meaning that it assigns higher probabilities to instances that are less likely to be positive (Figure 4). This could indicate a bias toward classifying negatives as potential positives, leading to overconfidence in low-probability predictions. Between 0.34 and 0.44, the predicted and observed values are relatively close, suggesting good calibration in this region. The model underestimates positive cases in the high-probability range (0.54–0.94), meaning it assigns lower probabilities to instances that are more likely positive. This is particularly evident in the 0.86 and 0.94 probability bins, where the actual fraction of positives reaches 100% and 98.67%, respectively, but the predicted probabilities are lower. This underconfidence could lead to missed true positives, making the model too conservative in assigning high probabilities. The Brier score is a key metric for assessing the performance of probabilistic models, particularly in calibration analysis. It measures the mean squared difference between predicted probabilities and actual binary outcomes, quantifying the accuracy of probability estimates. A lower Brier score indicates better calibration, meaning that the predicted probabilities more closely align with observed outcomes. In our analysis, the Brier scores for the SVM, RF, XGBoost, and MLP models were 0.10, 0.10, 0.11, and 0.09, respectively. These results suggest that the models exhibited good calibration, as reflected in their lower Brier scores.

### 2.4. Y-Randomization

**Figure 5:**
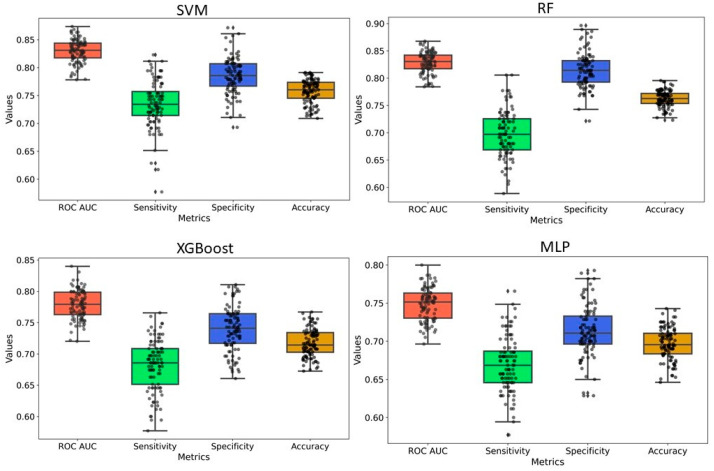
Y-randomization model for PaDEL+RDKit 2D descriptors-based model. For all models, 20% of the data were randomized, and the models were constructed using the same parameters as those applied to the original dataset.

**Figure 6:**
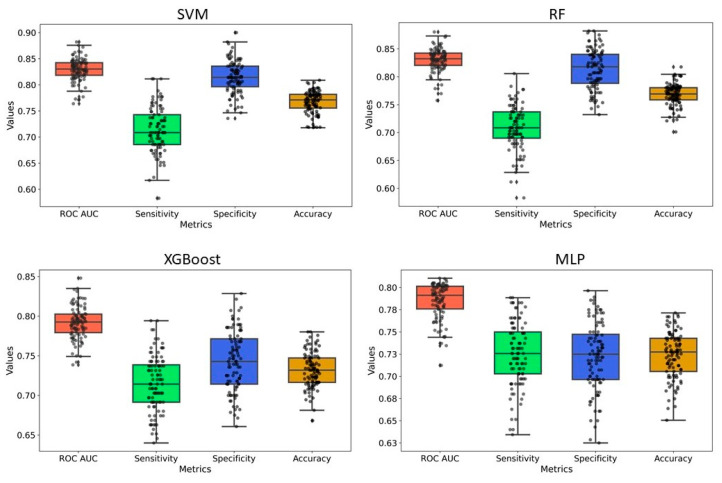
Y-randomization model for ECFP4 fingerprints-based model. For all models, 20% of the data were randomized, and the models were constructed using the same parameters as those applied to the original dataset.

Y-randomization was employed to assess the risk of obtaining classification models due to chance correlations. In our analysis, the average accuracy, sensitivity, and specificity of the randomized models were generally lower than those of the original model, with only a few exceptions. Furthermore, the AUC values for the randomized models were consistently lower, and none of the 100 random trials outperformed the original model (Figure 5 and Figure 6 and Supplementary Figures S2 and S3). These findings confirm that the predictive models developed in this study are robust and not the result of random chance. Therefore, the dataset and selected molecular descriptors demonstrate strong discriminatory power between active and inactive compounds.

### 2.5. Consensus Model

**Figure 7:**
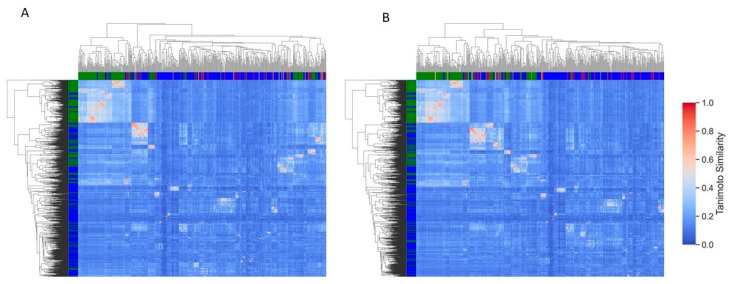
Consensus model’s prediction. This clustered heatmap represents the classification of compounds using a consensus model. (**A**) Prediction based on RDKit+PaDEL 2D descriptors. (**B**) Prediction based on ECFP4 fingerprints. Green color, True Positives; Blue color, True Negatives; Orange color, False Positives; Red color, False Negatives. Tanimoto Similarity has been calculated using the ECFP4 (bits = 1024) fingerprints. Row, Training Set compounds, Column, and Test Set compounds.

To overcome the limitations of a single model, we construct a consensus model that averages the predictions of all four models. A compound is considered accurately predicted if at least three out of four methods classify it correctly. This presents a comparison between the actual activity of compounds and their predicted activity (Consensus), along with a rank score for each compound. Higher rank scores indicate correct classifications, enabling the identification of promising compounds with greater confidence. This reduces the risk of false positives and enhances the efficiency of the screening process. The outcomes of this analysis indicate that most of the true active compounds were accurately predicted with high-ranking scores. Sixty-two and seventy-eight active compounds were classified correctly with a rank score above 0.90 using 2D descriptors and ECFP4 fingerprint-based models, respectively. Among these, the compounds CHEMBL563631, CHEMBL549592, CHEMBL550590, CHEMBL560322, CHEMBL552176, CHEMBL3310083, CHEMBL3360127, CHEMBL3360124, CHEMBL558641, CHEMBL560265, CHEMBL3310415, CHEMBL3360132, CHEMBL562760, CHEMBL244963, and CHEMBL242609 achieved rank scores above 0.95, reflecting a high level of confidence in their classification as active by both predictive models. Furthermore, 28 and 15 active compounds were misclassified as inactive by all four methods in the ECFP4 and 2D descriptor-based models, respectively. Notably, several of these misclassified compounds, including CHEMBL560387, CHEMBL2180481, CHEMBL261833, CHEMBL272419, CHEMBL456338, CHEMBL3582038, CHEMBL149893, CHEMBL3288713, CHEMBL211014, and CHEMBL1173779, exhibited rank scores below 0.1. Additionally, some compounds are predicted as active but with moderate confidence (Score = 0.3–0.5), indicating potential uncertainty in the model’s classification. These compounds may need further investigation as they represent borderline predictions. To further validate the predictions, a heatmap was constructed to cluster the outcomes of consensus predictions, where rows represent the training set and columns represent the test set. This analysis aimed to assess whether false positives or negative compounds exhibit any similarity with true positives or negative compounds from the training set. The results show that most of these falsely predicted compounds do not have significant similarity with training set compounds (Figure 7). Some falsely predicted positive compounds have activity values between 1020 nM and 1600 nM. These compounds show moderate structural similarity (greater than 0.60 or 0.80) to some active compounds in the training set, likely contributing to the model’s misclassification. This suggests that the model may be struggling to differentiate compounds with borderline activity and requires refinement to more accurately distinguish between true positives and false positives, particularly in cases where the activity is near the threshold. This highlights the need for further refinement of the model to improve its accuracy in distinguishing between true positives and false positives, particularly for compounds near the threshold of activity.

### 2.6. Clustering, Scaffold Analysis, and Activity Landscape Assessment

**Figure 8:**
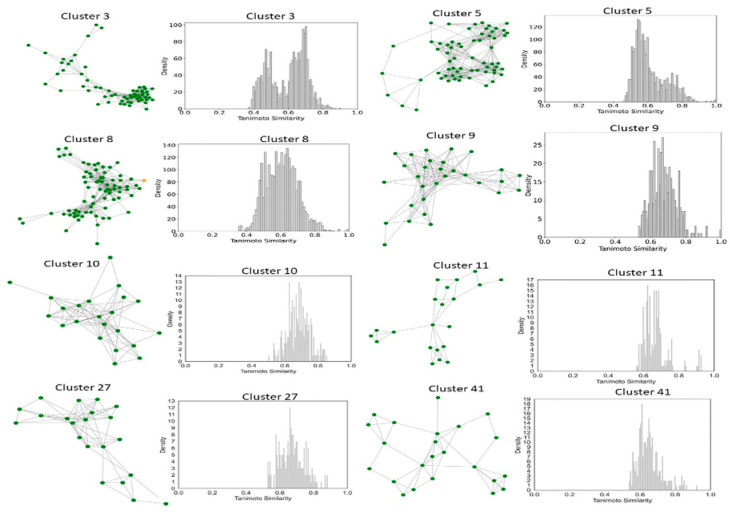
Compound Network and Tanimoto Coefficient Similarity Distribution for clusters enriched in active compounds. The network visualization represents the compounds within each cluster, with edges indicating pairwise Tanimoto coefficient similarities. The accompanying similarity distribution plot illustrates the range and frequency of Tanimoto coefficient values within the cluster, providing insights into the structural diversity of the compounds.

**Figure 9:**
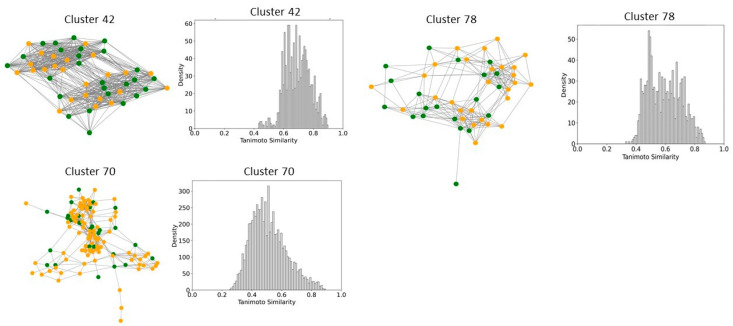
Compound Network and Tanimoto Coefficient Similarity Distribution for clusters enriched in mixed activity compounds. The network visualization represents the compounds within each cluster, with edges indicating pairwise Tanimoto coefficient similarities. The accompanying similarity distribution plot illustrates the range and frequency of Tanimoto coefficient values within the cluster, providing insights into the structural diversity of the compounds.

**Figure 10:**
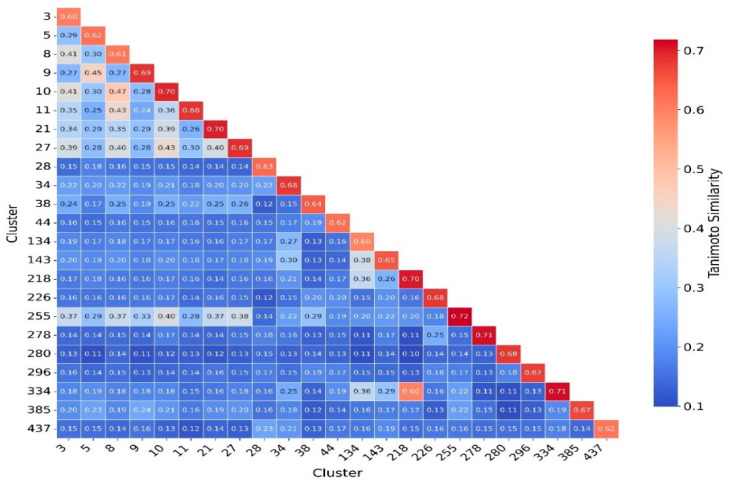
Cluster Similarity Heatmap Based on Tanimoto Coefficients. This heatmap illustrates the pairwise Tanimoto similarity between different molecular clusters based on their ECFP4 fingerprints. The color gradient represents the degree of similarity, with red indicating higher similarity and blue indicating lower similarity. Clusters are labeled on both axes, and numerical values within the cells indicate the average Tanimoto similarity between corresponding clusters. The color bar on the right provides a reference for interpreting similarity values.

To better understand and quantify significant structural classes associated with activity, we performed clustering analysis and analyzed the activity distribution within each cluster. A total of 756 clusters were identified using the NetworkX method, as described in the methods and materials. The majority of these clusters were small, consisting of only one or two compounds. Only 46 clusters contained 10 or more compounds. Among these, clusters such as 3, 5, 8, 9, 10, 11, 21, 27, 34, 38,41, 55, and 60 were found to be enriched in high-potency compounds, collectively comprising 374 compounds (Figure 8). For example, cluster 3 contains 61 compounds with activity ranging from 2 to 62 nM. Cluster 5 includes 64 compounds with activities ranging from 2 to 45 nM. Similarly, cluster 8 contains 75 compounds with activity spanning 3 to 130 nM. Clusters 9, 10, 11,21, and 27 contain 30, 22, 21,11, and 21 compounds, with activity distributions ranging from 20 to 28 nM, 3 to 26 nM, 3 to 18 nM, 7 to 30 nM, and 9 nM to 185 nM, respectively. In contrast, clusters 134, 197, 218, 226, 255, 278, 334, 335, 385, and 437 were enriched in low-potency compounds (Supplementary Figure S4). Clusters 42, 70, and 78 exhibited a mixed distribution of high- and low-potency compounds (Figure 9). Cluster 42 comprises 51 compounds with activity values ranging from 12 nM to 26,450 nM. Most inactive compounds in this cluster have activity values between 1020 nM and 9200 nM, whereas active compounds have activity between 12 nM and 820 nM. Similarly, Cluster 70 consists of 116 compounds with activity values ranging from 10 nM to 70,000 nM, with inactive compounds having activity values between 1080 nM and 9800 nM. This distribution suggests that the majority of inactive compounds fall within a moderate activity range. Since many of these compounds exhibit activity within a reasonable range, minor chemical modifications such as the introduction of functional groups or scaffold hopping could enhance their binding affinity and improve their pharmacological profiles. The heatmap generated from the Tanimoto similarity matrix offers valuable insights into the relationships and clustering patterns of the compounds (Figure 10 and Supplementary Figure S5). Compounds within clusters exhibit moderate structural similarity, with values ranging from 0.68 to 0.70 (Figure 10). This suggests that these clusters share common chemical scaffolds or functional groups. In contrast, other clusters contain structurally diverse compounds. Thus, the activity pattern in these clusters highlights the presence of highly potent compounds that, with structural modifications, could potentially be optimized into more potent derivatives.

**Figure 11:**
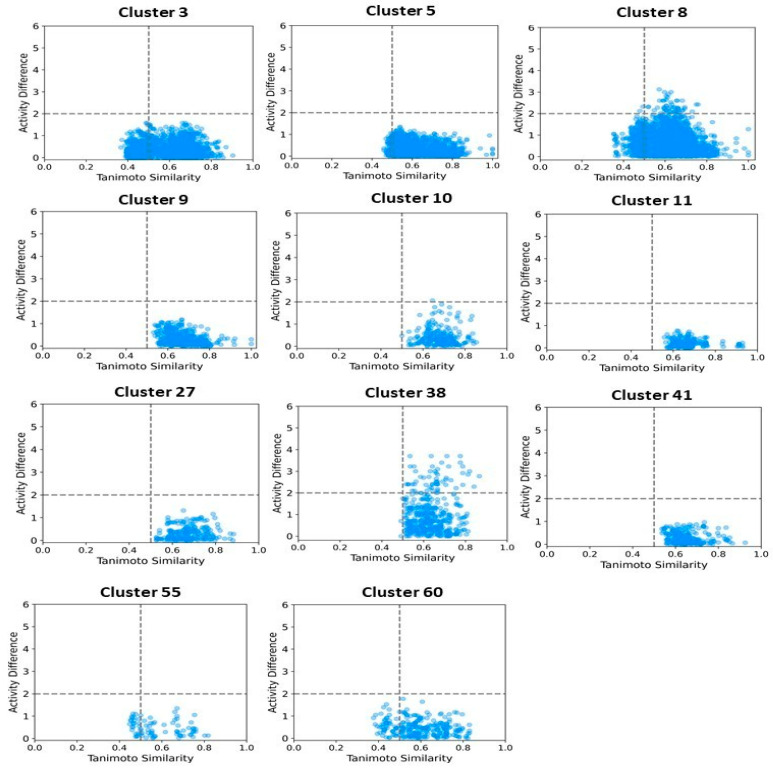
Activity landscape plot for clusters enriched in active compounds. The activity landscape visualizes the relationship between molecular similarity and activity differences within the cluster, highlighting regions of significant activity variation.

**Figure 12:**
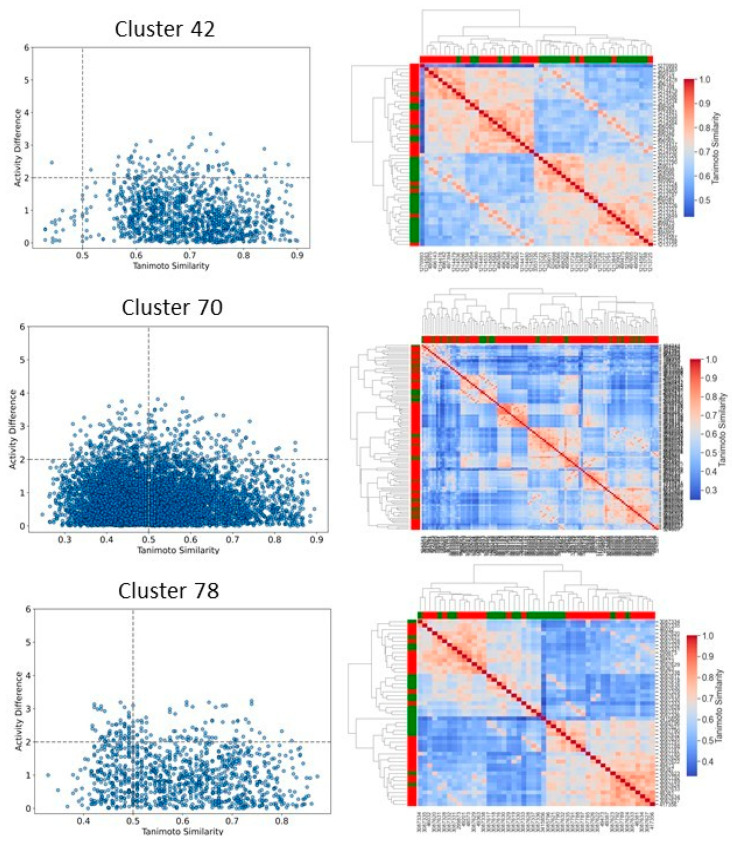
Activity landscape plot for clusters enriched in mixed compounds. The activity landscape visualizes the relationship between molecular similarity and activity differences within the cluster, highlighting regions of significant activity variation. The accompanying heatmap provides a detailed representation of the pairwise activity differences between compounds in the cluster, enabling the identification of trends and patterns in activity.

We then focused on clusters containing 10 or more compounds for activity divergence and scaffold analyses. For this analysis, we set a potency difference threshold of at least two log units and a similarity threshold of 0.5. As shown in Figure 11, clusters 3, 5, 9,10,11,27, 41,55, and 60 exhibited minimal activity variation, with the majority of chemical pairs being structurally diverse yet displaying low potency differences. In contrast, clusters 8 and 38, despite maintaining reasonable structural similarity, exhibited significant activity divergence. Clusters 42, 70, and 78 were identified as containing compounds with significant potency variations (Figure 12), where even minor structural modifications led to substantial differences in biological activity. For instance, compounds CHEMBL3582064, CHEMBL512195, CHEMBL3582071, and CHEMBL3582080 exhibited Tanimoto coefficient similarities above 0.75. Despite their high structural similarity, small chemical modifications led to significant potency changes. A specific example is the substitution of a chlorine (Cl) atom with a fluorine (F) atom, which resulted in an IC_50_ shift from 110 nM to 21,000 nM. Similarly, in cluster 42, compounds CHEMBL521567, CHEMBL1214417, CHEMBL1214480, and CHEMBL1214586 showed moderate Tanimoto coefficient similarities. However, minor structural modifications led to significant variations in their biological activity. In cluster 78, CHEMBL521567, CHEMBL1214480, CHEMBL1214417, and CHEMBL1214586 share Tanimoto similarities greater than 0.70. However, minor structural modifications—such as substituting chlorine (Cl) with fluorine (F)—resulted in a significant decrease in potency, increasing activity from 12 nM to 9200 nM. To gain deeper insights into the structural profiles and their associated activities, we performed a Bemis–Murcko scaffold analysis. Scaffolds like Pyrazolopyrimidine–benzene, Benzodiazolone–benzamide, Pyrrolopyridine–benzene, Pyrrolopyridine–benzamide, etc., are most prevalent in clusters that are enriched in active compounds. In contrast, clusters 42, 70, and 78, which have mixed activity compounds, include benzyl-substituted indole, benzylpyrrole, 1-benzyl-4-piperazinylquinolin-2-one, and 1-benzylindole. Further, we calculated N/M and N_sing_/M for each data set representing the scaffold diversity and comparison of scaffold novelty, and the results are shown in Table 3. The analysis of scaffold diversity and novelty across the clusters reveals key insights into their structural characteristics and potential utility in drug discovery. Clusters such as 38 and 41 exhibit high scaffold diversity (0.633 and 0.609, respectively) and novelty (0.600 and 0.565, respectively), making them promising candidates for exploring unique chemical spaces and identifying novel lead compounds. These clusters are likely to contain novel scaffolds that can expand the scope of chemical exploration. Similarly, moderately diverse clusters like 3, 10, and 27, with scaffold diversity scores ranging from 0.545 to 0.590 and novelty scores around 0.475 to 0.523, strike a balance between scaffold variety and innovation. These clusters may serve as valuable resources for further exploratory studies while maintaining a focus on discovering potential chemotypes. Conversely, clusters such as 28, 42, and 44 demonstrate minimal scaffold diversity and novelty, indicating highly homogeneous and redundant scaffold compositions. These clusters, while limited in the potential for innovative drug discovery, might still be helpful for targeted SAR studies or fine-tuning known chemical motifs. Clusters like 11 and 27, which exhibit high diversity (0.666 and 0.571, respectively) but relatively moderate novelty, reflect a rich variety of scaffolds with limited uniqueness. These clusters could be exploited to explore broad chemical libraries and generate insights into scaffold-driven biological activity. Some representative structures of common scaffolds from these clusters are provided in Supplementary Figure S6.

## 3. Discussion

We developed classification models to predict the probability that a given compound is an HIV Integrase inhibitor. These models were built using four different machine-learning methods and rigorously validated. Understanding the molecular features in drug design is essential, as they directly impact a compound’s interaction with biological targets such as enzymes, receptors, and other proteins. The ability to distinguish key molecular attributes helps optimize lead compounds, improving their efficacy, selectivity, and overall pharmacological profile. This insight is critical for guiding the rational design of novel therapeutic agents. The key features used in model building were selected using a hybrid approach, GA–SVM–RFE. Through feature importance analysis, we identified 44 key molecular descriptors that represented chemical properties related to topological, electrotopological, constitutional, and physicochemical aspects. This aligns with previous findings that descriptors related to topological, lipophilicity, and electrostatic properties play a significant role in drug design. Among these descriptors, the atom-type electrotopological state encodes information about the electronic state of bonded atoms within a compound and their topological context within the entire molecular structure [21]. This descriptor helps capture subtle electronic effects that influence molecular interactions. Similarly, the extended topochemical atom indices offer a detailed representation of the electronic environment surrounding atoms, bonds, functional groups, and branching patterns, providing a more comprehensive view of molecular characteristics [22]. EState_VSA4 and EState_VSA5 are molecular descriptors integrating Electrotopological State (E-State) indices with van der Waals surface area (VSA) measurements. These descriptors quantify the cumulative VSA of atoms within specific E-State index ranges, effectively capturing molecules’ electronic and steric properties. Through ANOVA analysis, the high F-scores of descriptors WTPT-5, fr_benzene, SM1_Dzi, maxsOH, VSA_EState9, etc., confirm their significant role in discriminating between active and inactive compounds, supporting their importance in capturing both electronic and steric properties. Descriptors related to topological and electrostatic features, such as SlogP_VSA3 and fr_benzene, further reinforce their critical contribution to molecular interactions and drug design. These findings align with the observed relevance of topological and lipophilicity properties in previous studies, highlighting their central role in optimizing molecular activity. The AD refers to the region in the vector space where a mathematical model, such as a QSAR model, can make reliable predictions, essentially representing the interpolation region [23]. For a new compound to be considered within the applicability domain, it must have enough similarity to the compounds in the training set used to build the model. In this study, to determine the AD, the Euclidean distance method was used [24]. Our results have shown that test set compounds fall within the region where the model has been trained, supporting the validity of using the model for these compounds. This finding reinforces the idea that the model is expected to provide accurate predictions for compounds within this range, ensuring its reliability within the defined AD. Simultaneously, the robustness of models was also examined using the Y-randomization test [25]. This method is widely recognized as one of the most rigorous validation procedures for QSAR models, providing a measure of their reliability and robustness [25,26]. Here, we performed a total of 100 randomization runs to evaluate the robustness of the developed models. As shown in Figure 5 and Figure 6, the Y-randomization test confirmed that the developed models did not exhibit random correlations, thereby demonstrating a genuine structure–activity relationship. An ideal model must provide precise predictions and have good calibration. In classification tasks, it is critical to not only predict the class label but also evaluate the probability of each class [27]. This probability reflects the confidence in the prediction. Some models may give poor estimates of class probabilities, and others may not provide probability predictions at all [28,29]. Well-calibrated models enable more precise threshold selection for classification decisions, facilitate better risk management, and enhance model generalization across different datasets [28]. This study uses a calibration plot to evaluate the model’s predictive accuracy, assessing how well its predicted probabilities align with the observed event rates. Figure 3 and Figure 4 show that the models demonstrate the agreement between predicted and actual outcomes. Additionally, the Brier score is used to estimate the model’s performance. The Brier score is a quadratic scoring function that calculates the squared difference between the actual binary outcomes and the predicted probabilities. For a model, the Brier score ranges from 0 (for a perfect model) to 1 (for a non-informative model). Our models showed lower Brier scores, suggesting better calibration and enhanced reliability in probability estimation.

The consensus model uses a combination of predictions and probabilities from multiple classifiers to produce a final prediction. While no explicit penalty term was added, this averaging approach inherently reduces the impact of extreme or uncertain predictions from any single model. In this sense, the method indirectly accounts for inconsistency by balancing outputs across multiple models. This approach improved the robustness of the predictions, primarily when individual models provided marginal or conflicting outputs [30].

By utilizing an equal-weight averaging strategy for prediction scores, the consensus method effectively balanced the contributions of multiple models, thereby reducing the impact of individual models or descriptor set limitations [31]. Here, we combine the outcomes of all four models to determine the final prediction for each compound. By calculating rank scores, we obtain a confidence measure for each compound, which is used to rank or select compounds for further analysis. The model was highly confident in predicting some of the active compounds with high-ranking scores, which highlights its effectiveness in accurately identifying promising candidates. Interestingly, some of the active compounds were found to have high-ranking scores from both models. This integrated approach leveraged the strengths of each model and descriptor set, resulting in more robust and accurate predictions [32].

The clusters identified in this study necessitate further examination to ascertain common substructures for derivative design utilizing medicinal chemistry and SAR data. Targeting HIV integrase requires creating and producing pharmaceuticals derived from clusters abundant in highly potent compounds. In medicinal chemistry and drug design, the concept of a ‘scaffold’ serves as a crucial framework for identifying, evaluating, and comparing key structural motifs within bioactive compounds and their analogs, thereby facilitating the rational design and discovery of new active molecules [33,34,35]. This process involves identifying scaffolds derived from existing activity data and an in-depth analysis of a library of derivative compounds to identify those that exhibit optimal potency and selectivity [36]. These scaffolds represent chemically significant entities that may offer opportunities for discovering new biologically relevant scaffold classes [37]. This study aimed to identify and compare scaffolds, assessing their diversity across significant clusters. We applied the Murcko scaffold method [38], which decomposes a molecule into its core ring system by preserving all rings and the linkers between them while removing the side chains. From this study, we identified scaffolds like Pyrazolopyrimidine–benzene, Benzodiazolone–benzamide, Pyrrolopyridine–benzene, and Pyrrolopyridine–benzamide scaffolds, most prevalent in clusters that are enriched in active compounds. These scaffolds are commonly seen in molecules with excellent binding affinity to the catalytic core domain of HIV integrase [39,40,41]. The pyrazolopyrimidine core offers heteroatoms that are capable of forming hydrogen bonds and coordinating with magnesium ions in the active site of HIV integrase. The pyrazolo[1,5-a] pyrimidine (PP) core is a fused, rigid, and planar N-heterocyclic system comprising both pyrazole and pyrimidine rings [42]. This fused pyrazole is considered a privileged scaffold in combinatorial library design and drug discovery owing to its exceptional synthetic flexibility [43]. The benzene ring adds hydrophobic interactions with the enzyme’s binding pocket. The benzodiazolone core allows for π–π stacking interactions and hydrogen bonding, which is critical for stabilizing the inhibitor in the active site. The benzamide group enhances the compound’s hydrophobic and polar interactions within the enzyme pocket. Pyrrole derivatives are involved in many biological processes and find pharmacological applications as anticancer [44], antibacterial [45], and HIV [39]. Development is due to their diverse interaction mechanisms with the enzyme. The pyrrolopyridine core facilitates π–π interactions and provides multiple sites for hydrogen bonding [46,47]. This scaffold is frequently observed in compounds demonstrating strong binding affinity to the catalytic core domain of HIV integrase [48]. Scaffold diversity is a critical aspect of drug discovery, as structurally diverse scaffolds provide a broader chemical space for lead optimization and enhance the potential for identifying novel bioactive compounds [49]. A chemically diverse set of scaffolds within a cluster can enhance the chances of identifying potent drug candidates by exploring different binding interactions with the target. In contrast, similar scaffolds may share key features, which help in optimizing lead compounds. The scaffold diversity/novelty of HIV compounds in clusters was computed and compared. Table 1 summarizes the number of compounds (M), Bemis–Murcko scaffolds (N), and singleton scaffolds (Ns) present in each cluster, offering insights into the structural heterogeneity of the dataset. The ratio of Ns to scaffolds (Nss/Ns) provides more information on the distribution of molecules over scaffolds. Evaluating scaffold diversity within clusters allows researchers to balance novelty and similarity, which is significant for identifying structurally diverse and pharmacologically relevant candidates. The high scaffold diversity and novelty observed in clusters 38 and 41 suggest their potential for expanding the chemical space. Moderately diverse clusters, such as 3, 10, and 27, offer a balanced combination of scaffold variety and novelty. This finding shows that such clusters may serve as valuable sources for identifying structurally novel lead compounds with promising pharmacological properties. Overall, the exploration and comparison of scaffolds from the selected clusters allowed us to identify key scaffolds and chemotypes that could contribute to designing new compound libraries and developing drug candidates for HIV treatment.

## 4. Materials and Methods

### 4.1. Data Preparation

The datasets employed for model construction were extracted from the ChEMBL database [50]. Among the tested compounds, those that exhibited an activity of 1 µM were reported as active, whereas all remaining compounds were reported as inactive. The dataset was divided into training and test sets in a ratio of 80:20 for model development and validation.

### 4.2. Molecular Descriptors and Selections

To characterize the structural features of the compounds, a comprehensive set of molecular descriptors was generated using PaDEL-Descriptor [51] and RDKit [52]. We employed a hybrid approach combining Genetic Algorithms (GA) and Support Vector Machine Recursive Feature Elimination (SVM-RFE) [53] for feature selection to identify the most relevant descriptors for modeling the biological activity of compounds. The GA–SVM–RFE method operates by first using a GA to evolve a population of binary vectors, where each vector represents a potential subset of descriptors [54]. The SVM-RFE process ranks the selected features according to their importance for distinguishing between active and inactive compounds, removing the least important ones iteratively. The fitness of each individual is determined by the cross-validated accuracy of the SVM model trained on the selected features. The GA optimizes the feature subset by selecting, mating, and mutating individuals to converge toward an optimal set of descriptors. The GA used a population size of 20 individuals and ran for 10 generations, with a crossover probability of 0.5 and a mutation probability of 0.2. We also employed ANOVA F-score analysis to evaluate the discriminative power of each of the selected 44 descriptors. The ANOVA F-score measures the variance between classes (active vs. inactive) for each feature, providing a statistical basis for interpreting each descriptor. A higher F-score indicates a greater ability to distinguish between active and inactive compounds. These features with high F-scores contribute significantly by highlighting the most informative descriptors, which enhance the model’s ability to classify compounds based on their biological activity correctly.

### 4.3. Model Building

In this study, four different machine learning methods were used to develop the predictive models, namely, SVM [55], Random Forest (RF) [56], XGBoost [57], and multiple linear perceptions (MLP) [58] using the scikit-learn Python module 3.7. The regularization parameter (C) for the SVM was set to 6. Additionally, the radial basis function (RBF) kernel was used, with the gamma parameter set to 0.001. Random Forest is an ensemble learning method that constructs multiple decision trees, each trained independently on a randomly chosen subset of the data. It is widely used in cheminformatics for its effectiveness in handling high-dimensional datasets with small sample sizes while maintaining robustness against overfitting. The RF Classifier method with balanced class weights was used to build the model. Grid search was performed using 50, 75, 100, and 200 estimators with the AUC as a scoring function. XGBoost is an advanced ensemble learning algorithm, well-suited for classification tasks due to its ability to handle large datasets, manage missing values, and provide robust performance on high-dimensional data. For this study, XGBoost was employed to develop classification models, leveraging its efficiency and effectiveness in producing accurate predictions. Model training and hyperparameter tuning were performed to optimize classification performance. Model training was conducted to enhance performance, with hyperparameters set as follows: learning rate = 0.01, number of estimators (n_estimators) = 10, and maximum tree depth max_depth = 5. The Multi-Layer Perceptron is a feedforward artificial neural network designed for supervised learning tasks, including classification and regression. This study employed the MLP classifier to develop a classification model. The architecture of the MLP consisted of a single hidden layer with 100 neurons (hidden_layer_sizes = 100), leveraging the ReLU activation function. This model was trained using the Adam optimizer (solver = ‘adam’) with an initial learning rate of 0.00005 (learning_rate = 0.00005). The training was performed over 1000 iterations (max_iter = 1000) with a batch size of 16 (batch_size = 16).

### 4.4. Robustness of Models

To evaluate the statistical significance of our predictive models, we utilized the Y-randomization approach [25]. The robustness of the training set models was assessed by comparing them to models built from randomly permuted datasets. These datasets were generated by randomly shuffling the target property values among the training set compounds while maintaining their original value range. If the original model’s AUC lies above the 95th percentile of the randomized AUC scores, it is considered statistically significant, indicating that the model performance is unlikely due to chance. This procedure disrupts genuine structure–property relationships, serving as a control to verify the reliability and predictive power of the original QSPR models.

### 4.5. Model Evaluation

The predictive model was validated following the criteria established by the OECD [59]. Statistical metrics, including accuracy, recall, precision, and F1 score, were used to evaluate model quality. In addition, the model’s performance was assessed using a Receiver Operating Characteristic (ROC) curve [60] and the area under the curve (AUC). Predictions were generated for both the training and test set compounds.

$$Recall=\frac{TP}{TP+FN}$$

$$Precision=\frac{TP}{TP+FP}$$

$$\;\;\;\;\;\;\;\;\;F1=2\times \frac{Precision\times Recall}{Precision+Recall}$$

$$Accuracy=\frac{TP+TN}{TP+FP+FN+TN}$$

$$BrierScore=\frac{1}{N}\sum_{a=1}^{N}{\left(f_{a}-O_{a}\right)}^{2}$$

where *f_a_* is the probability that was forecast; *O_a_* is the actual outcome of the event at the instance, and *N* is the number of compounds.

### 4.6. Consensus Modeling

**Figure 13:**
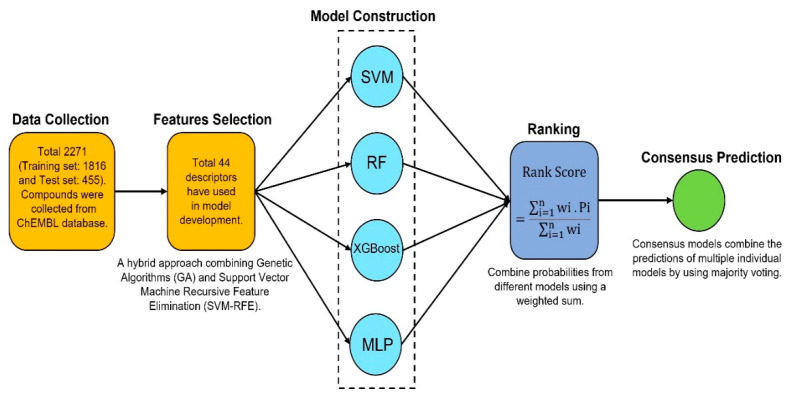
Flowchart for consensus model development.

This model combines the predictions of multiple individual models by using majority voting to determine the final prediction for each sample [61,62]. The final prediction is based on the outcomes from the four individual models: RF, SVM, XGB, and MLP. Figure 13 represents the workflow of the consensus model development. The predictions from each model (RF, SVM, XGB, and MLP) are collected for every sample. If at least three out of four models predict the compound as active (class 1), then the consensus model will predict that compound as active. Otherwise, it will predict that the compound is inactive (class 0). This model calculates a weighted average of the predicted probabilities for each class (active) from all the models. The weights for each model are set to be equal (1 for each model, in this case). Additionally, a penalty term is included in the final score, which is based on the variance of predictions across the models. This penalty helps reduce the influence of compounds where the models disagree significantly. The predicted probabilities for each class from each model are averaged using a weighted sum. A penalty is added based on the variance in the predictions of the models.

$$Rank\;Score=\frac{\sum_{i=1}^{n}wi\cdot Pi}{\sum_{i=1}^{n}wi}$$

where Pi is the probability predicted by model I, and wi is the weight of model i.

### 4.7. Clustering Analyses

This study utilized molecular fingerprinting, network analysis, and community identification methods to categorize chemicals based on their structural similarities. The compounds were represented by ECFP fingerprints [63], calculated using the Morgan algorithm with a radius of 2 and 1024 bits. To assess structural similarity, we computed the Tanimoto similarity between pairs of compounds. A similarity threshold of 0.75 was applied to determine significant similarities, and edges were created between compounds with similarity values above this threshold. A graph was constructed where nodes represented compounds, and edges represented substantial structural similarities. Community detection was performed using the Louvain method [64,65], identifying clusters of compounds with similar structures. Visualization was performed by plotting the generating community network, where nodes were colored based on their activity (Active or Inactive).

### 4.8. Scaffold and Structural Activity Variations

An extensive scaffold analysis was performed utilizing Murcko scaffolds [38], with calculations for each substance carried out on the RDKit platform. To investigate significant structural changes affecting biological activity, we identified activity cliffs—combinations of compounds with high structural similarity but marked differences in activity levels. This process entailed conducting a similarity search in which each active compound was compared to all inactive compounds and vice versa. Pairs with a moderate-to-high Tanimoto coefficient (Tc) similarity were identified as activity cliffs. While previous studies used thresholds ranging from 0.5 to 0.55 for comparable analyses, we chose a Tc cut-off of 0.5 to strengthen our findings [66].

Scaffold diversity=N/M

Here, N is the total number of scaffolds in the dataset, while M represents the total number of molecules. A higher N/M ratio indicates greater scaffold diversity.

$$Scaffold\;novelty=N_{sing}/M$$

Here, $N_{sing}$ is the number of singleton scaffolds, which are scaffolds that appear in only one dataset (“sing” denotes “singleton”), while M represents the total number of molecules in the dataset.

### 4.9. Compound Similarity

All similarity values presented in this study are based on the Tanimoto coefficient, calculated using Morgan fingerprints [63] with a radius of 2 and 1024 bits. The Tanimoto coefficient ranges from 0 (no similarity) to 1 (perfect similarity).

## 5. Conclusions

In this study, we developed predictive models for predicting the activity of HIV-1 integrase inhibitors using four different machine-learning methods and 44 molecular descriptors. The robustness of these models was thoroughly assessed through calibration and Y-randomization approaches, ensuring their reliability. The validated models demonstrated exceptional performance in accurately predicting the activity of compounds. Consensus prediction for each compound was determined by combining the outputs of all four models using majority voting. The Rank score proved to be a reliable indicator of correct predictions. Most of the highly active compounds were accurately ranked by both the 2D and ECFP4-based models, highlighting their effectiveness in identifying potent inhibitors. The consensus modeling approach demonstrated advantages over individual QSAR models by better balancing classification performance and predictive reliability. The SAR analysis pinpointed key scaffolds and functional groups contributing to HIV-1 integrase inhibition, offering insights into critical molecular activity determinants. Additionally, activity landscape analyses revealed significant SAR heterogeneity, with several compounds exhibiting high activity divergence, where even minor structural modifications led to substantial variations in biological activity. Cluster analysis identified significant classes associated with potent activity, with some clusters enriched in highly potent compounds while maintaining moderate scaffold diversity. These findings highlight promising candidates for exploring unique chemical spaces and identifying novel lead compounds. Integrating multiple machine learning methods helps mitigate the biases and limitations of any single model. This strategy enhances the overall accuracy and generalizability of the predictions, making the models more reliable in predicting the activity of HIV-1 integrase inhibitors across a diverse range of compounds. In conclusion, consensus modeling, extensive descriptor sets, and robust validation techniques offer a robust framework for developing reliable predictive models, thereby making it a significant contribution to HIV-1 integrase inhibitor research.

## Figures and Tables

**Table 1:** Performance of models using the selected descriptor set.

Methods	Data Set	Accuracy	Sensitivity	Specificity	Precision	F1
SVM	Training Set	0.88	0.83	0.91	0.87	0.85
	Test Set	0.87	0.81	0.90	0.84	0.83
RF	Training Set	0.97	0.97	0.98	0.98	0.97
	Test Set	0.89	0.80	0.95	0.92	0.85
XGB	Training Set	0.98	0.97	0.98	0.98	0.98
	Test Set	0.88	0.81	0.93	0.87	0.84
MLP	Training Set	0.97	0.97	0.98	0.97	0.96
	Test Set	0.87	0.81	0.91	0.85	0.83

**Table 2:** Performance of models using ECFP4 (bits = 1024) fingerprints.

Methods	Data Set	Accuracy	Sensitivity	Specificity	Precision	F1
SVM	Training Set	0.87	0.78	0.92	0.88	0.83
	Test Set	0.86	0.77	0.92	0.85	0.81
RF	Training Set	0.90	0.84	0.94	0.91	0.87
	Test Set	0.87	0.78	0.92	0.86	0.82
XGB	Training Set	0.90	0.85	0.95	0.93	0.89
	Test Set	0.85	0.77	0.91	0.85	0.80
MLP	Training Set	0.92	1.00	0.99	1.00	1.00
	Test Set	0.84	0.66	0.93	0.86	0.74

**Table 3:** Scaffold diversity analysis for significant clusters.

Cluster	N	Ns	Nss	Ns/N	Nss/N
3	61	36	29	0.59	0.47
5	64	20	12	0.31	0.18
8	75	4	19	0.05	0.25
9	30	8	6	0.26	0.20
10	22	12	11	0.54	0.50
11	21	14	7	0.66	0.33
27	21	12	9	0.57	0.42
28	29	19	18	0.65	0.62
38	30	19	18	0.63	0.60
41	23	14	13	0.60	0.56
42	51	1	0	0.02	0.00
44	15	1	0	0.06	0.00
70	116	10	2	0.09	0.02
78	48	1	0	0.02	0.00

N, number of compounds; Ns, number of scaffolds; Nss, number of single scaffolds.

## Data Availability

Data is contained within the article..

## Supplementary Materials

The following supporting information can be downloaded at: https://www.mdpi.com/article/10.3390/ph18050714/s1, Figure S1. Applicability Domain Plot. (A) Based on RDKit+PaDEL 2D descriptors. (B) Based on ECFPs fingerprints. Figure S2. Y-Radomerization model. For all models, 20% of the data were randomized, and the models were constructed using the same parameters as those applied to the original dataset. Figure S3. Y-Radomerization model for ECFPs. For all models, 20% of the data were randomized, and the models were constructed using the same parameters as those applied to the original dataset. Figure S4. Compound Network for significant clusters. Figure S5. Tanimoto Coefficient Similarity Distribution for compounds in significant clusters. Figure S6. List of the most common scaffolds identified within each cluster. Table S1. List of slected significant descriptors.
