# Supplementary material for: Machine Learning-Driven Consensus Modeling for Activity Ranking and Chemical Landscape Analysis of HIV-1 Inhibitors
Source: Pharmaceuticals (Basel). 2025 May 13;18(5):714. doi: 10.3390/ph18050714 (PMC12115078; doi:10.3390/ph18050714)
Supplement: Supplementary file 1 [file pharmaceuticals-18-00714-s001.zip › Supplementary Figures.pptx]

## Slide 1
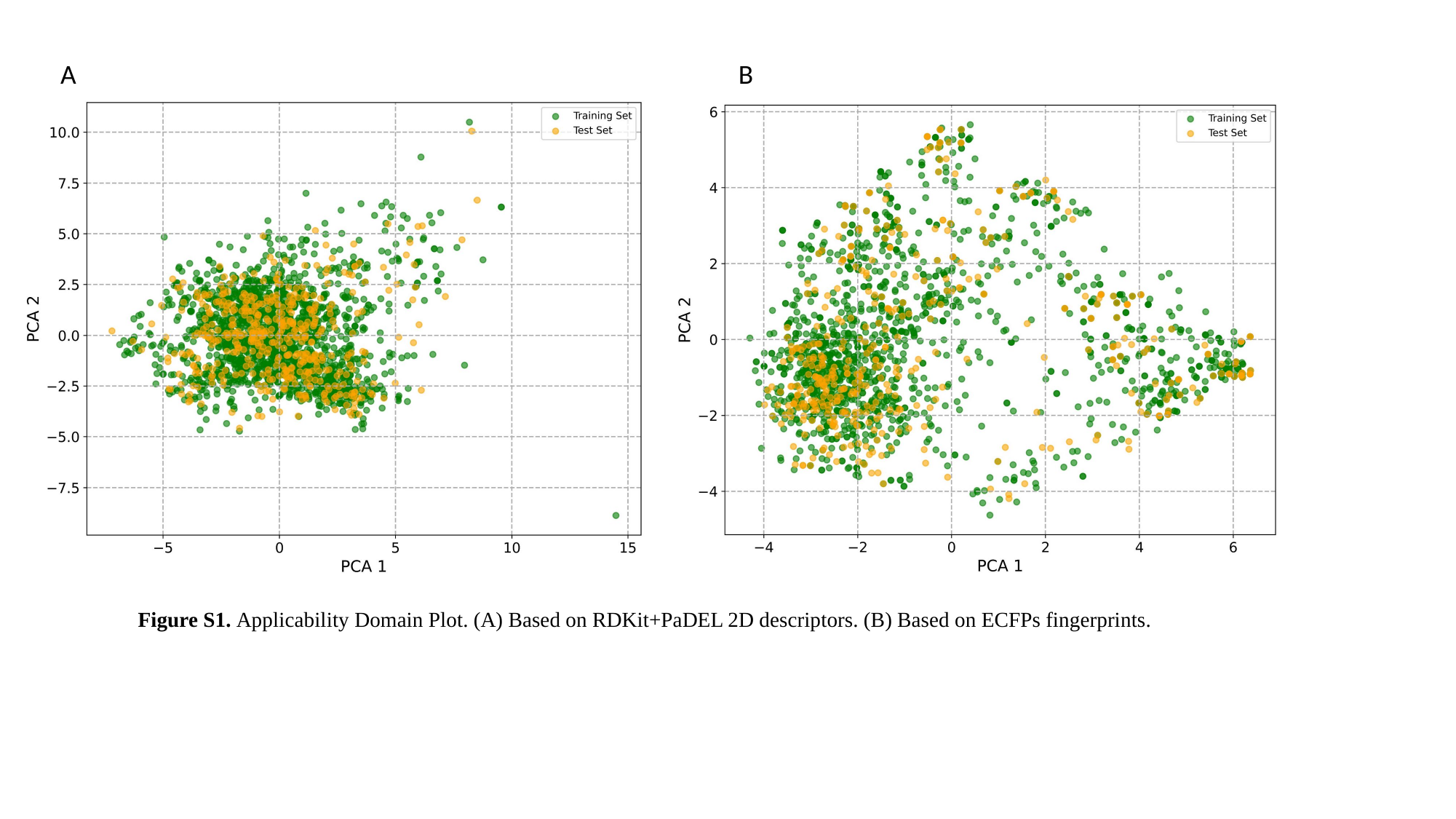

A
B
Figure S1. Applicability Domain Plot. (A) Based on RDKit+PaDEL 2D descriptors. (B) Based on ECFPs fingerprints.

## Slide 2
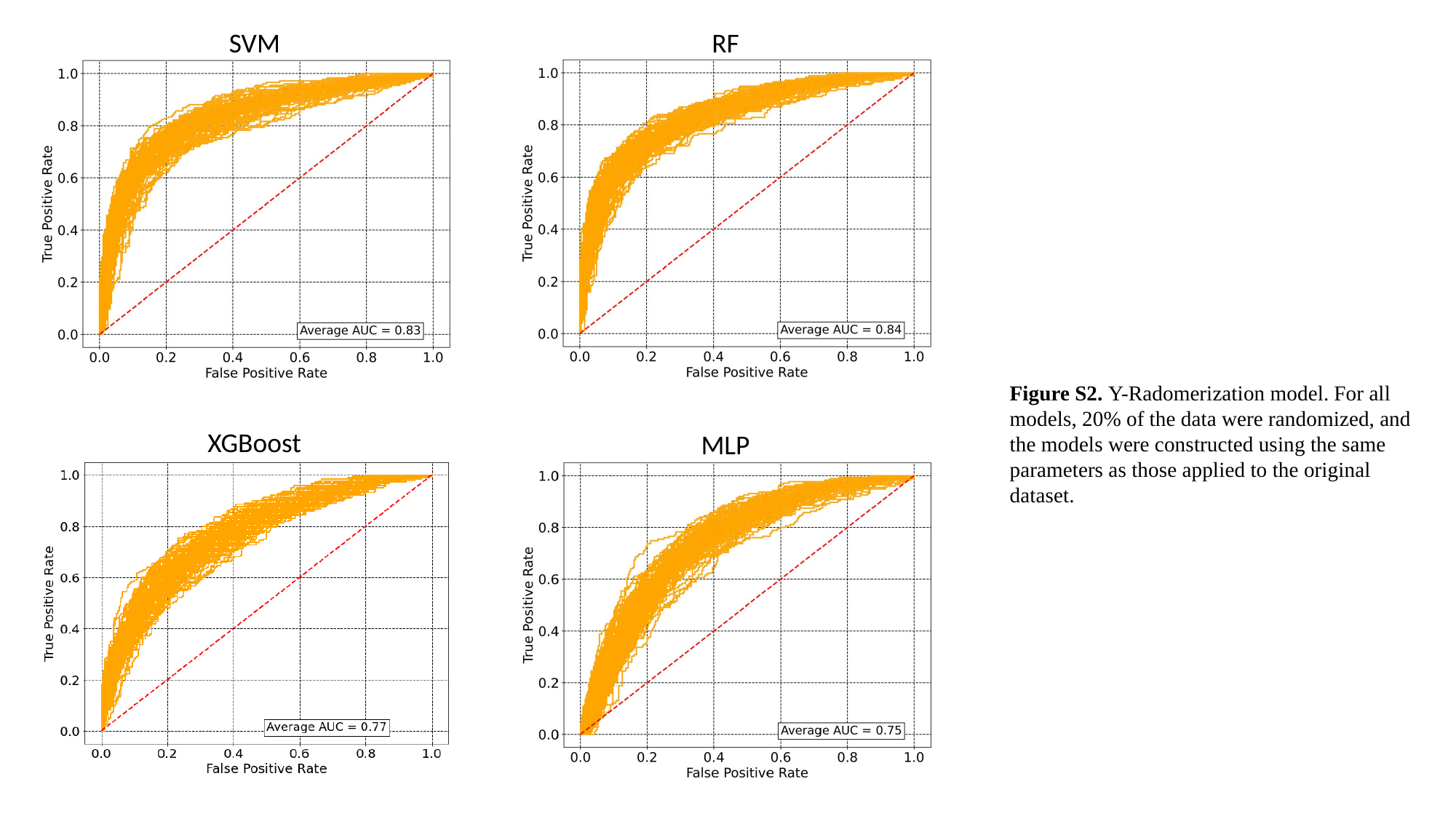

RF
SVM
XGBoost
MLP
Figure S2. Y-Radomerization model. For all models, 20% of the data were randomized, and the models were constructed using the same parameters as those applied to the original dataset.

## Slide 3
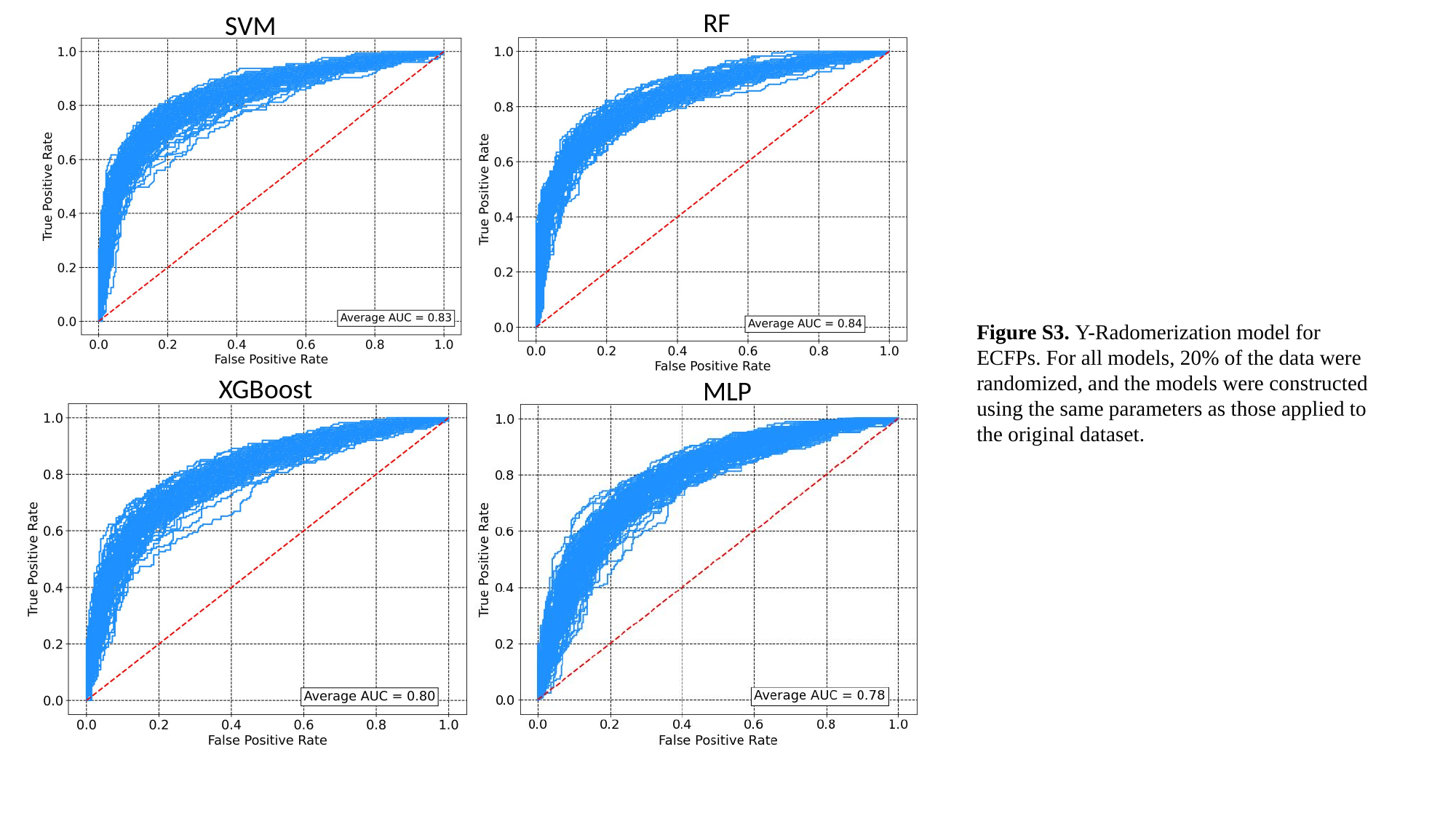

RF
SVM
XGBoost
MLP
Figure S3. Y-Radomerization model for ECFPs. For all models, 20% of the data were randomized, and the models were constructed using the same parameters as those applied to the original dataset.

## Slide 4
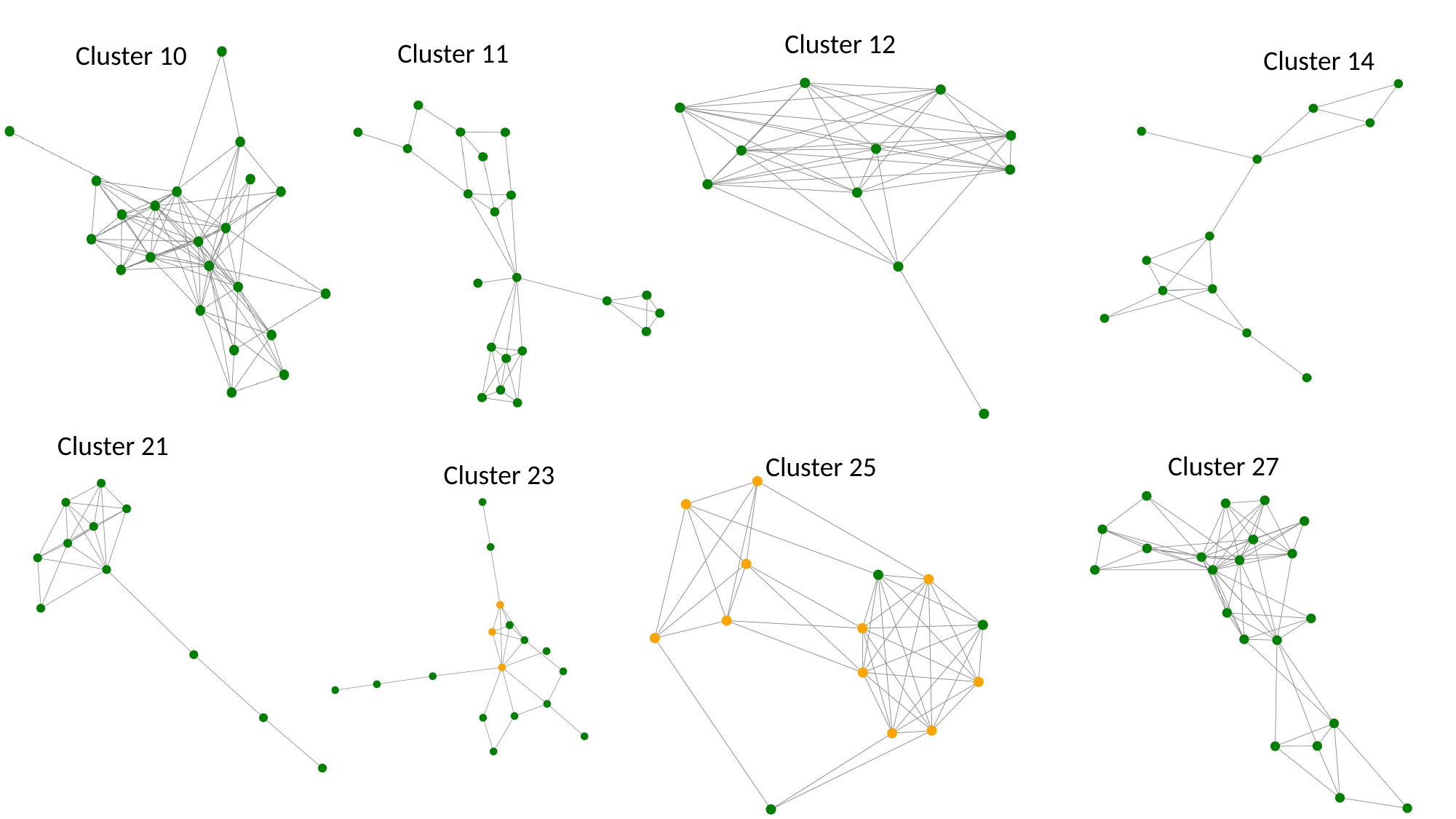

Cluster 12
Cluster 11
Cluster 10
Cluster 21
Cluster 27
Cluster 25
Cluster 23
Cluster 14

## Slide 5
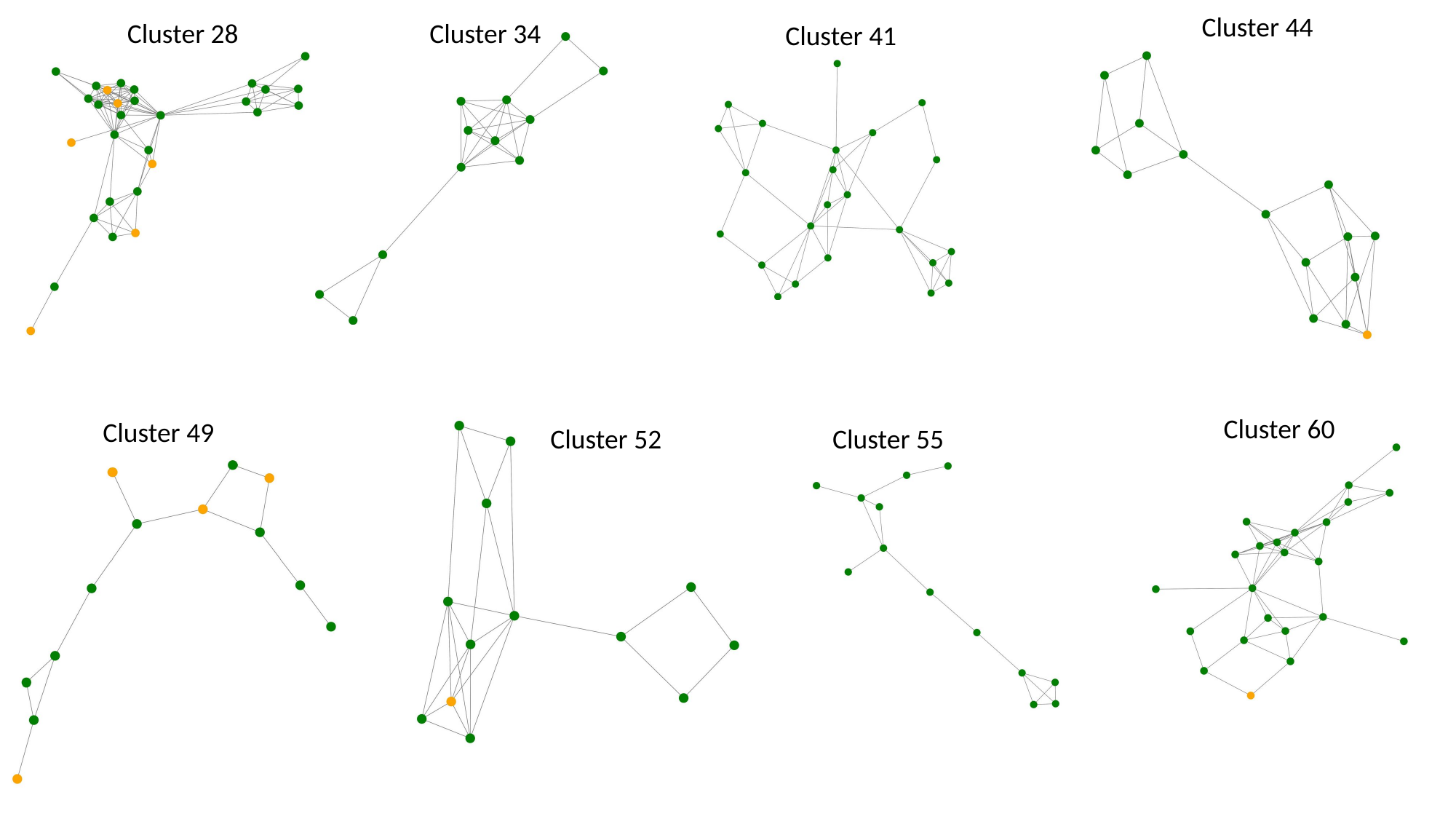

Cluster 44
Cluster 28
Cluster 34
Cluster 41
Cluster 60
Cluster 49
Cluster 52
Cluster 55

## Slide 6
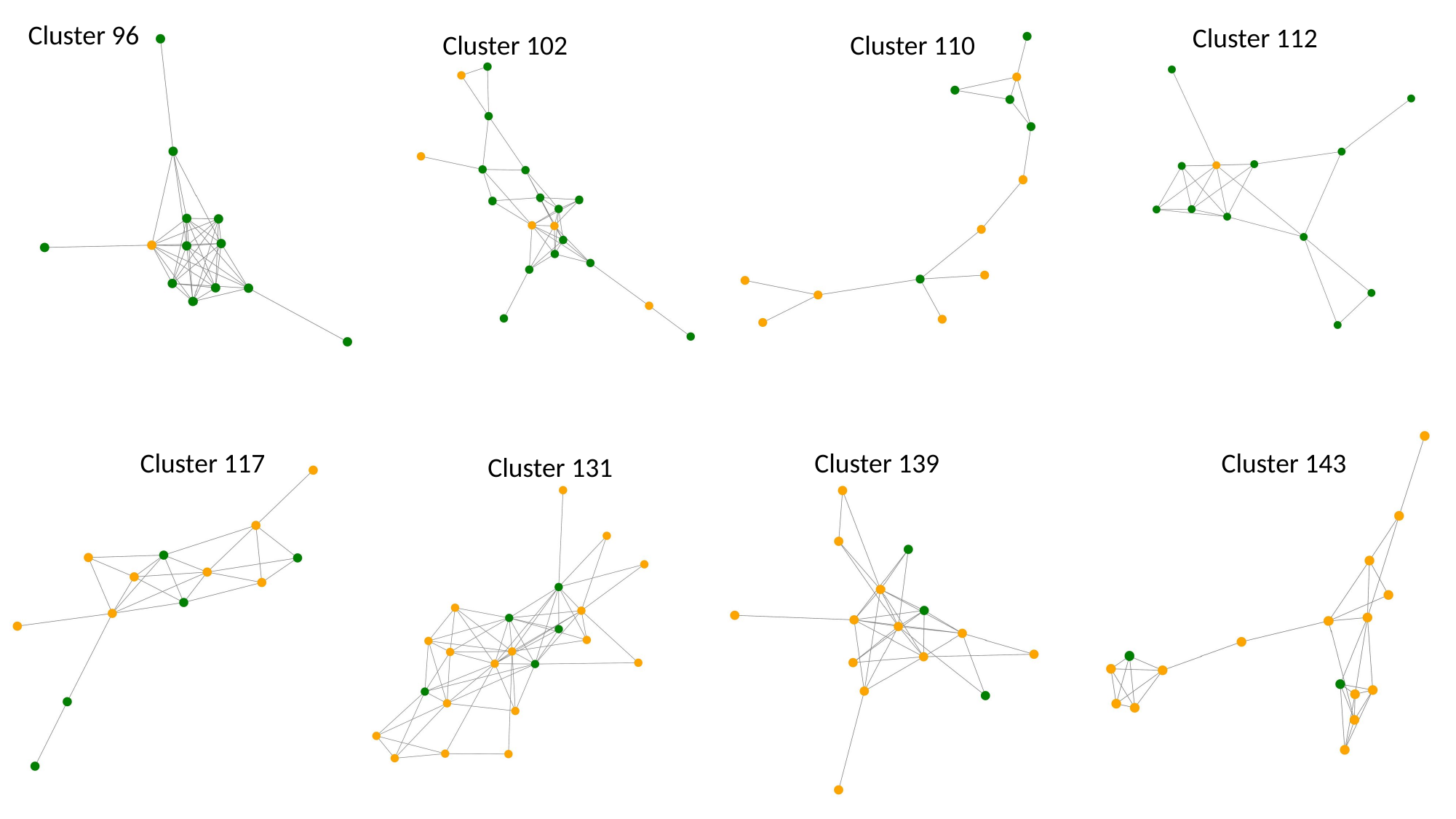

Cluster 96
Cluster 112
Cluster 102
Cluster 110
Cluster 143
Cluster 117
Cluster 139
Cluster 131

## Slide 7
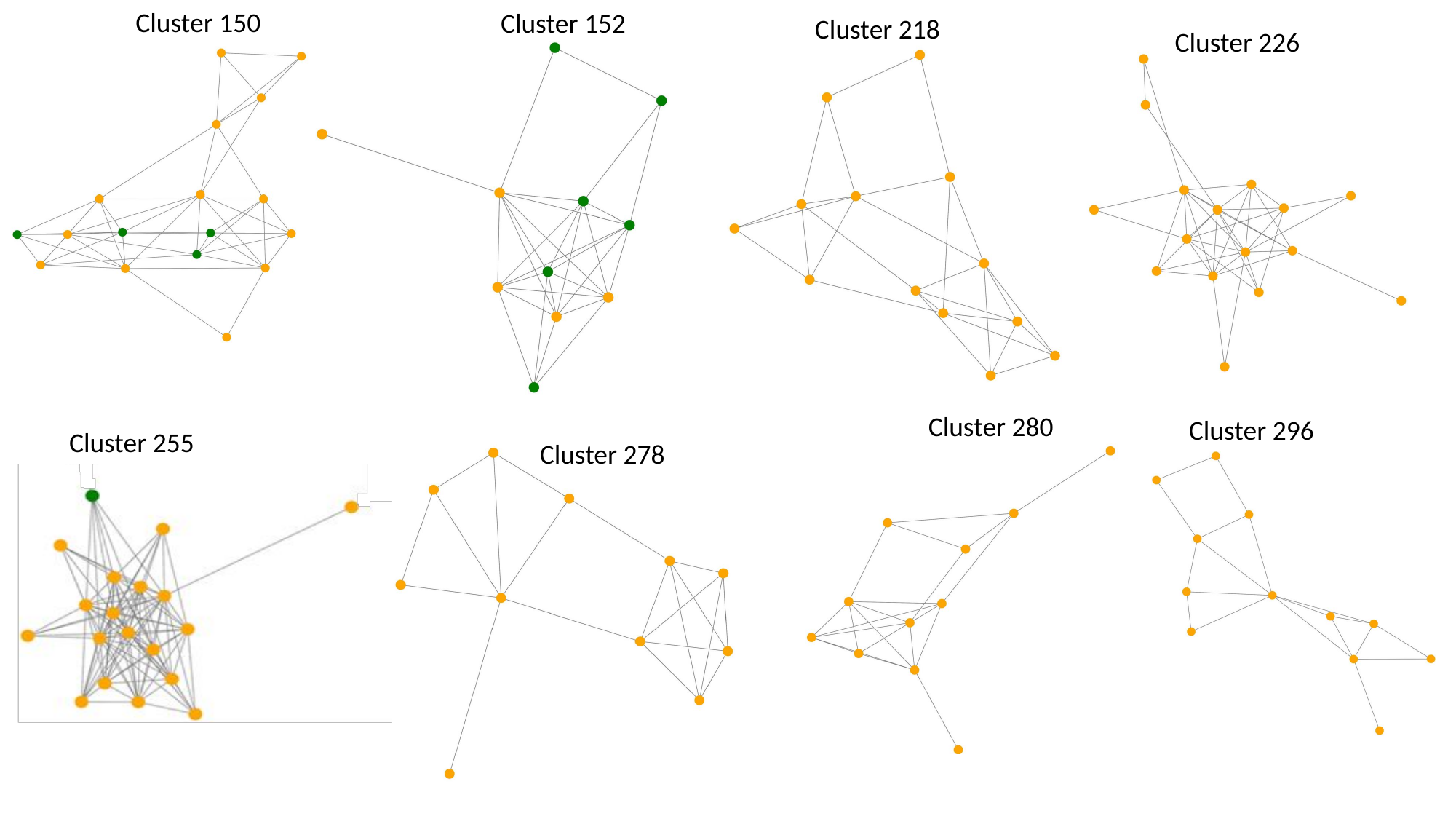

Cluster 150
Cluster 152
Cluster 218
Cluster 226
Cluster 280
Cluster 296
Cluster 255
Cluster 278

## Slide 8
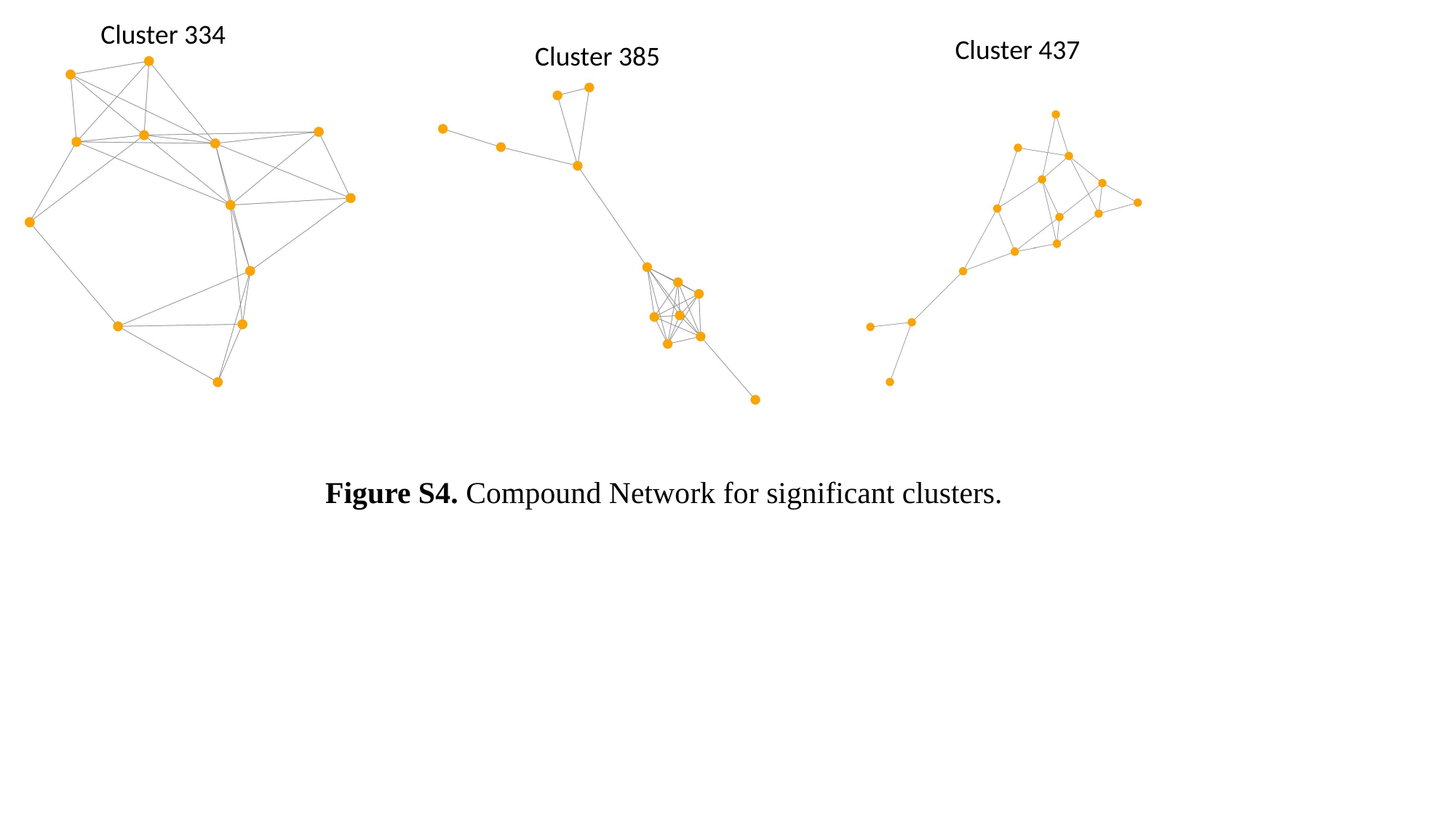

Cluster 334
Cluster 437
Cluster 385
Figure S4. Compound Network for significant clusters.

## Slide 9
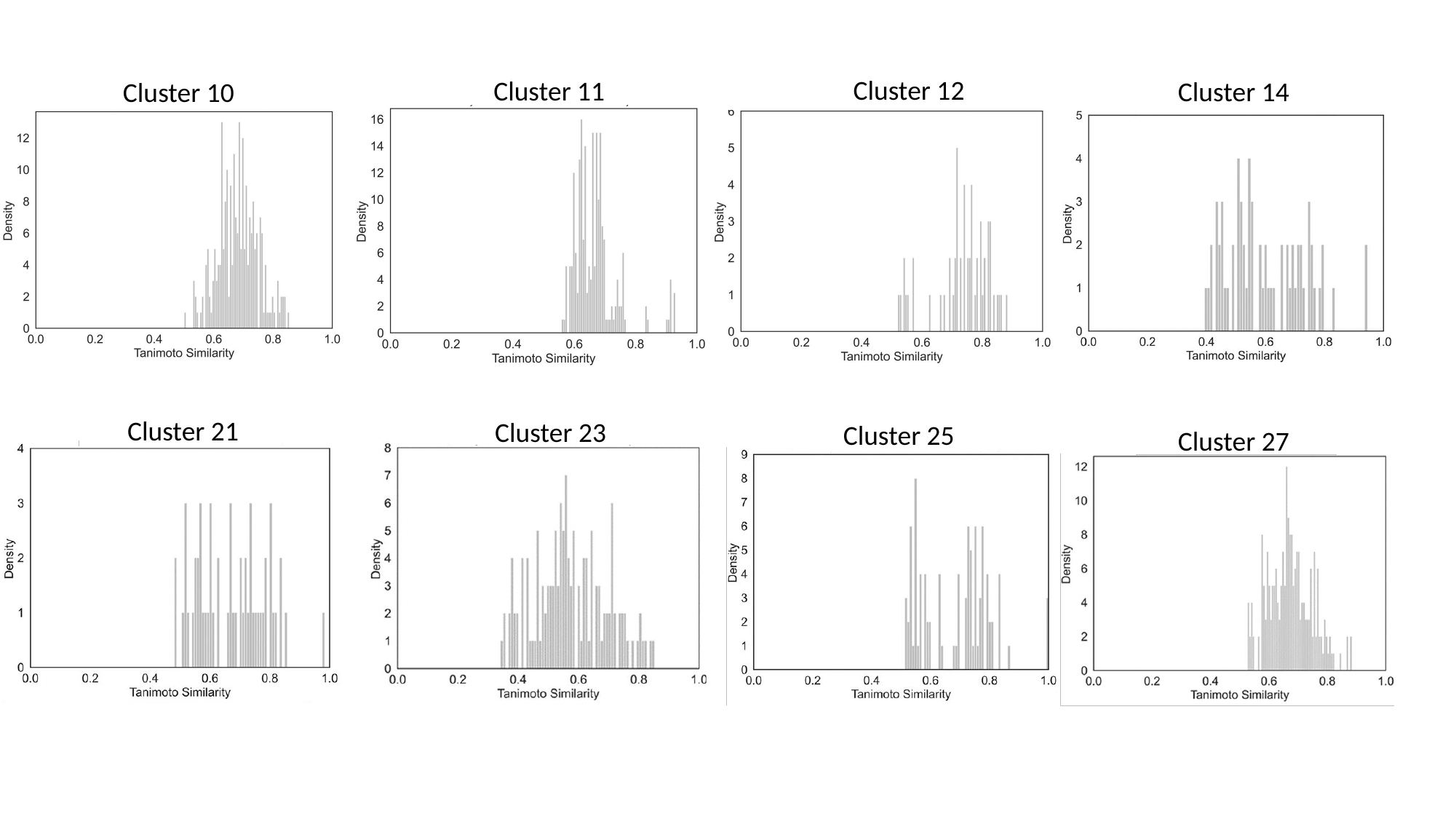

Cluster 12
Cluster 11
Cluster 14
Cluster 10
Cluster 21
Cluster 23
Cluster 25
Cluster 27

## Slide 10
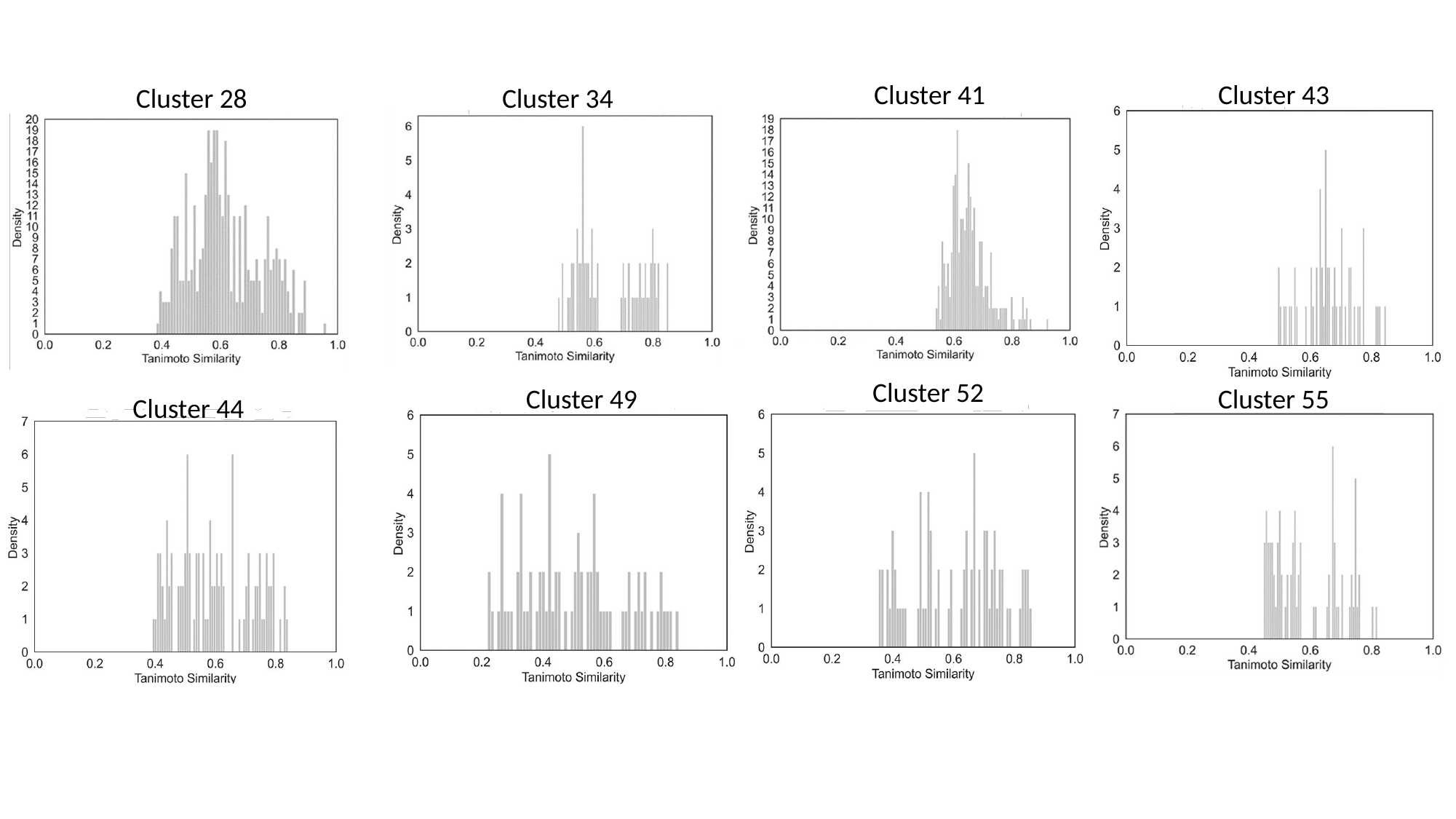

Cluster 41
Cluster 43
Cluster 28
Cluster 34
Cluster 52
Cluster 49
Cluster 55
Cluster 44

## Slide 11
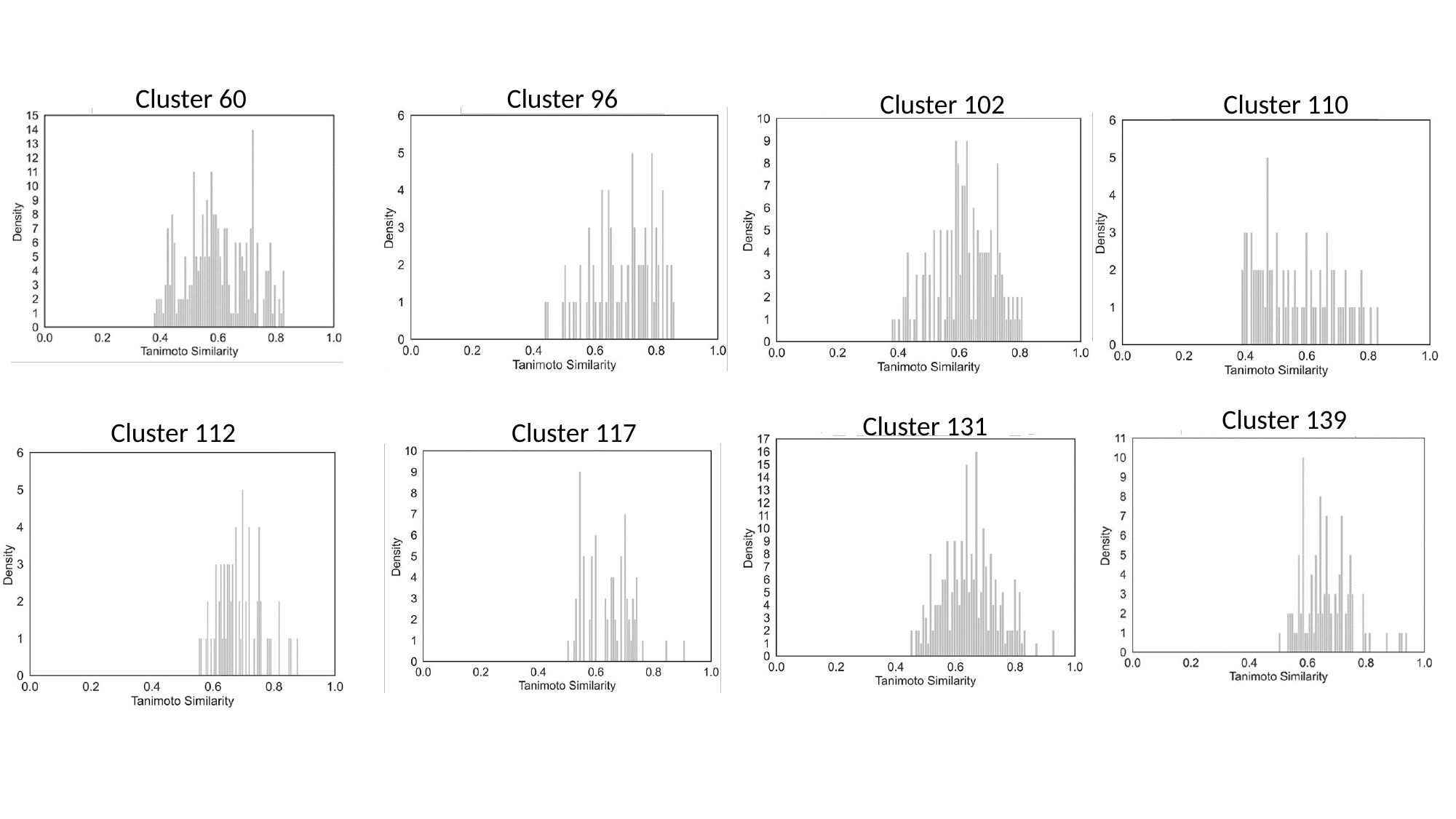

Cluster 60
Cluster 96
Cluster 102
Cluster 110
Cluster 139
Cluster 131
Cluster 112
Cluster 117

## Slide 12
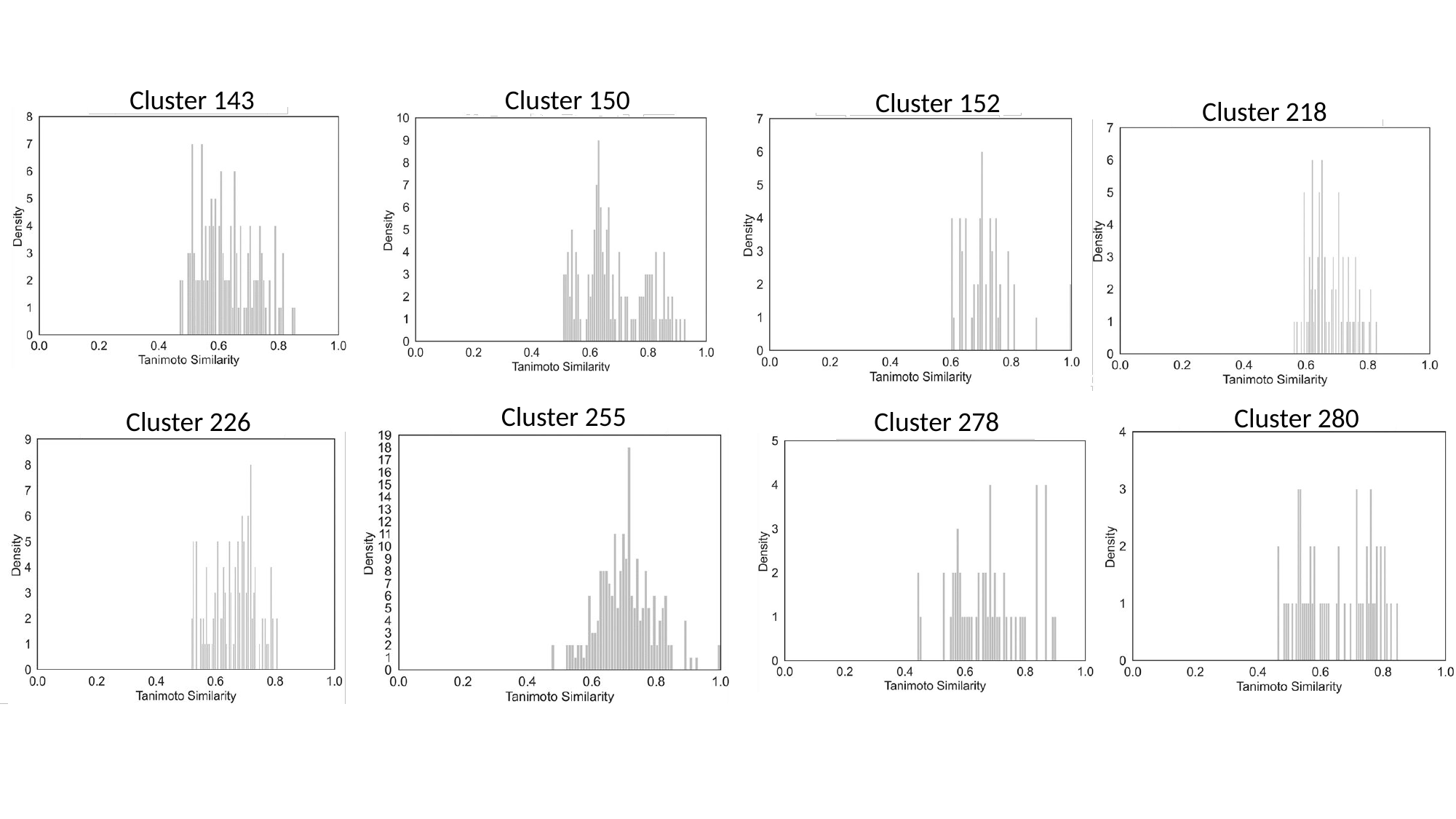

Cluster 143
Cluster 150
Cluster 152
Cluster 218
Cluster 255
Cluster 280
Cluster 226
Cluster 278

## Slide 13
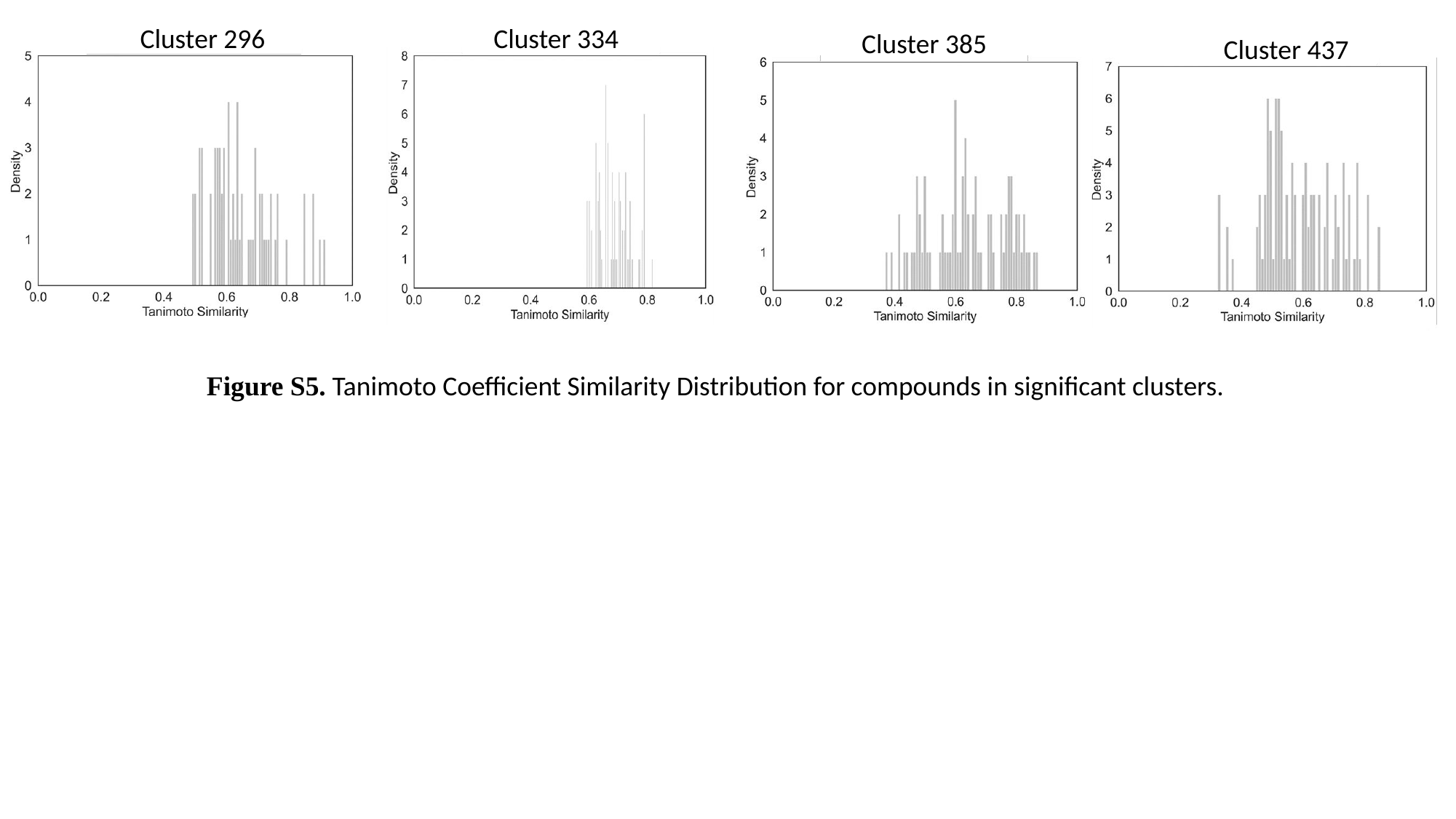

Cluster 296
Cluster 334
Cluster 385
Cluster 437
Figure S5. Tanimoto Coefficient Similarity Distribution for compounds in significant clusters.

## Slide 14
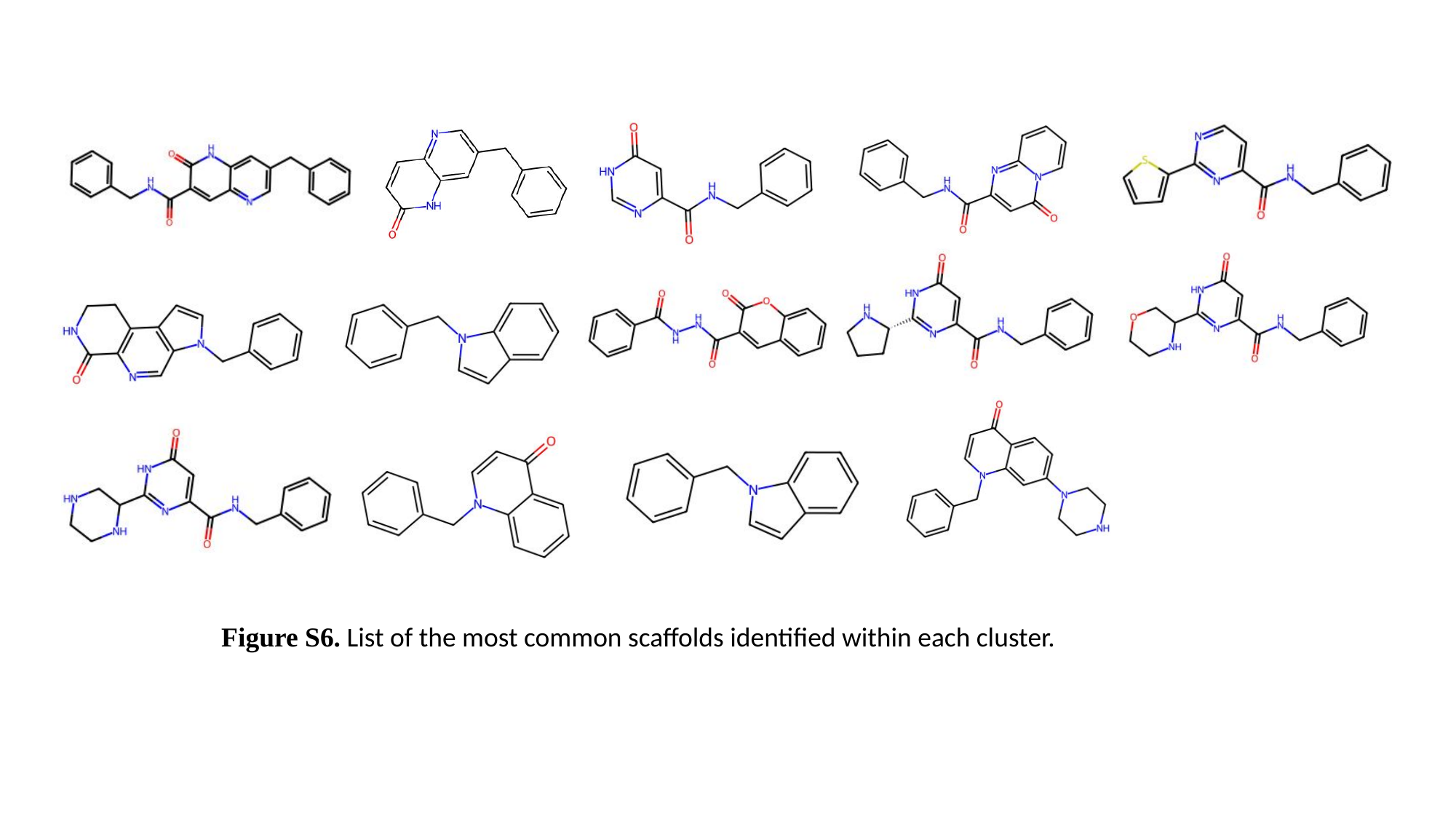

Figure S6. List of the most common scaffolds identified within each cluster.
