# Supplementary material for: Machine Learning-Driven Consensus Modeling for Activity Ranking and Chemical Landscape Analysis of HIV-1 Inhibitors
Source: Pharmaceuticals (Basel). 2025 May 13;18(5):714. doi: 10.3390/ph18050714 (PMC12115078; doi:10.3390/ph18050714)
Supplement: Supplementary file 1 [file pharmaceuticals-18-00714-s001.zip › Supplementary_Table.pdf]

Table S1. List of slected significant descriptors.

Descriptor	Name	Total count
ALOGP	ALogp2	1
Autocorrelation	ATS3s, MATS6c, MATS8c, MATS2m, MATS4p, MATS2s, GATS3v, GATS2i, GATS2s, GATS4s	10
Barysz matrix	SM1_Dzi, VE3_Dzi, SM1_Dzs, VE2_Dzs	4
Carbon types	C3SP2	1
Chi chain	VCH-5	1
Constitutional	Mv,	1
Burden modified eigenvalues	Mi	1
Atom type electrotopological state	nsBr, SHBint2, SdsCH, minHBa, maxHBint3, maxHBint8, maxdNH, maxsOH, maxssO, maxsCl	10
Extended topochemical atom	ETA_EtaP_F	1
Information content	CIC0	1
Molecular distance edge	MDEC-22	1
Weighted path	WTPT-4, WTPT-5	2
	BCUT2D_LOGPHI	1
Number of carboxylic acids	fr_COO2	1
Number of benzene rings	fr_benzene	1
The number of Nitrogens and Oxygens	NOCount	1
EState	EState_VSA4, EState_VSA5, VSA_EState5, VSA_EState9	4
Molecular surface area descriptors	SlogP_VSA3	1
Ring	nT8Ring	1
